# ZNF212 promotes genomic integrity through direct interaction with TRAIP

**DOI:** 10.1093/nar/gkac1226

**Published:** 2023-01-03

**Authors:** Hee Jin Chung, Joo Rak Lee, Tae Moon Kim, Soomi Kim, Kibeom Park, Myung-Jin Kim, Eunyoung Jung, Subin Kim, Eun A Lee, Jae Sun Ra, Sunyoung Hwang, Ja Yil Lee, Orlando D Schärer, Yonghwan Kim, Kyungjae Myung, Hongtae Kim

**Affiliations:** Department of Biological Sciences, Ulsan National Institute of Science and Technology, Ulsan 44919, Republic of Korea; Department of Biological Sciences, Ulsan National Institute of Science and Technology, Ulsan 44919, Republic of Korea; Department of Biological Sciences, Ulsan National Institute of Science and Technology, Ulsan 44919, Republic of Korea; Center for Genomic Integrity Institute for Basic Science (IBS), Ulsan 44919, Republic of Korea; Department of Biological Sciences, Ulsan National Institute of Science and Technology, Ulsan 44919, Republic of Korea; Department of Biological Sciences, Ulsan National Institute of Science and Technology, Ulsan 44919, Republic of Korea; Department of Biological Sciences, Research Institute of Women's Health and Digital Humanity Center, Sookmyung Women's University, Seoul 04310, Republic of Korea; Department of Biological Sciences, Research Institute of Women's Health and Digital Humanity Center, Sookmyung Women's University, Seoul 04310, Republic of Korea; Department of Biological Sciences, Ulsan National Institute of Science and Technology, Ulsan 44919, Republic of Korea; Center for Genomic Integrity Institute for Basic Science (IBS), Ulsan 44919, Republic of Korea; Center for Genomic Integrity Institute for Basic Science (IBS), Ulsan 44919, Republic of Korea; Center for Genomic Integrity Institute for Basic Science (IBS), Ulsan 44919, Republic of Korea; Department of Biological Sciences, Ulsan National Institute of Science and Technology, Ulsan 44919, Republic of Korea; Department of Biological Sciences, Ulsan National Institute of Science and Technology, Ulsan 44919, Republic of Korea; Center for Genomic Integrity Institute for Basic Science (IBS), Ulsan 44919, Republic of Korea; Department of Biological Sciences, Research Institute of Women's Health and Digital Humanity Center, Sookmyung Women's University, Seoul 04310, Republic of Korea; Department of Biological Sciences, Ulsan National Institute of Science and Technology, Ulsan 44919, Republic of Korea; Center for Genomic Integrity Institute for Basic Science (IBS), Ulsan 44919, Republic of Korea; Department of Biological Sciences, Ulsan National Institute of Science and Technology, Ulsan 44919, Republic of Korea; Center for Genomic Integrity Institute for Basic Science (IBS), Ulsan 44919, Republic of Korea

## Abstract

TRAIP is a key factor involved in the DNA damage response (DDR), homologous recombination (HR) and DNA interstrand crosslink (ICL) repair. However, the exact functions of TRAIP in these processes in mammalian cells are not fully understood. Here we identify the zinc finger protein 212, ZNF212, as a novel binding partner for TRAIP and find that ZNF212 colocalizes with sites of DNA damage. The recruitment of TRAIP or ZNF212 to sites of DNA damage is mutually interdependent. We show that depletion of ZNF212 causes defects in the DDR and HR-mediated repair in a manner epistatic to TRAIP. In addition, an epistatic analysis of Zfp212, the mouse homolog of human ZNF212, in mouse embryonic stem cells (mESCs), shows that it appears to act upstream of both the Neil3 and Fanconi anemia (FA) pathways of ICLs repair. We find that human ZNF212 interacted directly with NEIL3 and promotes its recruitment to ICL lesions. Collectively, our findings identify ZNF212 as a new factor involved in the DDR, HR-mediated repair and ICL repair though direct interaction with TRAIP.

## INTRODUCTION

The Eukaryotic genome is continuously exposed to various genotoxic insults originating from exogenous or endogenous sources (1,2). Genomic instability and cell death can ensue if such DNA lesions are not properly repaired. To maintain genomic integrity, cells utilize delicate DNA damage response (DDR) signaling and DNA repair pathways (3,4). A particularly dangerous class of lesions is DNA interstrand crosslinks (ICLs), which block DNA strand separation, thereby inhibiting several vital DNA metabolic processes including DNA replication and transcription. Unrepaired ICLs can cause DNA breaks and lead to chromosomal rearrangements. The repair of ICLs requires genes from multiple DNA repair pathways, including HR, translesion synthesis (TLS), and nucleotide excision repair (NER) and can occur in a replication-dependent or -independent manner (5–7). Defects in ICL repair genes are associated with chromosome instability syndromes, including Fanconi anemia (FA). FA is a rare human genetic disease characterized by bone marrow failure, developmental abnormalities, and cancer predisposition (8,9). FA is caused by mutations in at least 23 independent genes. FA cells are hypersensitive to DNA crosslinking agents, such as mitomycin C (MMC) and cisplatin (10–12) and show increased chromosomal breaks and radial chromosomes in response to these drugs (13–16). At least three pathways for the repair of ICLs have been identified in *Xenopus* egg extracts that differ in how the ICL is unhooked upon being encountered by the replication fork: in the first one, the replicative DNA helicase CMG is unloaded upon the convergence of two replication forks at an ICL (17). This pathway involves the polyubiquitylation of MCM7 and its subsequent unloading by p97 (18). Removal of CMG complex leads to fork reversal, activation of the FA pathway and unhooking of the ICL by nucleolytic incisions and eventual repair of the resulting DSB by HR (19,20). The second pathway utilizes the NEIL3 DNA glycosylase to cleave one of two *N*-glycosyl bonds of the ICL, leaving an abasic site on one template strand that is bypassed by TLS polymerases. Recently, a third pathway for ICL repair was reported for acetaldehyde-induced ICLs (21). This pathway involves the direct reversal of the ICL without any strand incisions by an unknown factor and is dependent on the Y-family DNA polymerase REV1 to complete repair of ICLs. Even though all three pathways are activated by encounter of the fork with the ICLs, the FA pathway is the only on that involves CMG unloading. Mutations in FA genes lead to more severe sensitivity to crosslinking agents than mutations in *NEIL3* (22,23), suggesting that cells likely first utilize the NEIL3 pathway, but depend more heavily on the FA pathway for cell survival. Furthermore, the NEIL3 pathway only acts on certain ICLs, including those formed by psoralen and abasic sites in *Xenopus* egg extracts.

The Ring-type E3 ligase TRAIP (TRAF (tumor necrosis factor receptor-associated factor)-interacting protein), also known as RNF206, was shown to be essential for embryonic development in Drosophila (encoded by NOPO (no pole)) and mice (24,25). TRAIP was shown to associate with replication forks encountering ICLs *in vitro* (26). The depletion of TRAIP led to hypersensitivity to MMC and increased MMC-induced radial chromosome formation (27). TRAIP appears to function as a master regulator that modulates the choice of ICL repair pathways by regulating the status of MCM7 ubiquitination in *Xenopus* egg extracts (17). In mammals, TRAIP plays important roles in the DDR and replication-associated HR-mediated DNA repair to maintain genomic integrity (17,27–31). Mutations in *TRAIP* were also found in patients with primordial dwarfism (29) and the patient mutation (R18C) was defective for MCM7 polyubiquitylation and repairing ICL lesions in *Xenopus* egg extracts (17). Furthermore, TRAIP was proposed to regulate both the NEIL3 and FA pathways for repairing psoralen-ICLs in human cells (32). Altogether, previous reports suggested that TRAIP is a versatile DDR and DNA repair factor involved in the response to a various types of DNA damage. However, how TRAIP modulates the damage signaling, HR and ICL repair is not fully understood.

In this study, we identified ZNF212 as a novel interacting partner of TRAIP and showed that it targets TRAIP to DNA lesions to promote HR-mediated DNA repair. In ICL repair, Zfp212, the mouse homolog of ZNF212, appears to act upstream Neil3 and Fanconi anemia (FA) pathways in the same way as Traip does. ZNF212 stimulates the repair of TMP-ICLs by interacting with NEIL3, promoting its localization to ICL lesions. Overall, our study shows that the TRAIP-ZNF212 axis is important for the DNA damage signaling, HR and ICL repair in mammalian cells.

## MATERIALS AND METHODS

### Cell culture

U2OS, HeLa, and human embryonic kidney (HEK) 293T cell lines were purchased from American Type Culture Collection (Manassas, VA). The cell lines were maintained in Dulbecco modified Eagle's medium (DMEM; Invitrogen, Carlsbad, CA) supplemented with 10% fetal bovine serum (FBS; Gibco, Franklin Lakes, NJ) and 1% antibiotic/antimycotic (Gibco) in 5% CO_2_ in a 37°C incubator. For mouse ES culture, AB2.2 mouse ES (mES) cells were maintained in M15 medium containing leukemia inhibitory factor (LIF): high-glucose Dulbecco's modified Eagle's medium (DMEM) supplemented with 15% fetal bovine serum, 100 μM mercaptoethanol, 1 mM glutamine, 3 mg/ml penicillin, 5 mg/ml streptomycin, and 1000 U/ml ESGRO leukemia inhibitory factor (LIF). These cells were grown on 0.1% of gelatin-coated plastic plate in 5% CO^2^ in a 37°C incubator (33).

### Plasmids

DNA primers used for PCR, cloning, and sequencing were obtained from macrogen (Seoul, South Korea) and listed in the Supplementary Table S1. The GFP-TRAIP and Myc-TRAIP, TRAIP D-1, D-2, D-3, D-4 and D-5 deletion mutant expression plasmids were previously described (34,35). ZNF212 gene was purchased from the Korea Human Gene Bank. Green fluorescence protein (GFP)-tagged ZNF212, ZNF212 D-1, D-2, D-3, D-4, D-5, D-6 and D-7 deletion mutant expression plasmids were cloned into green fluorescence protein (GFP)-tagged mammalian expression vector using DNA primers described in Supplementary Table S1. The SFB-tagged ZNF212 expression plasmid was cloned into SFB (S-tag, Flag epitope, and streptavidin-binding peptide)-tagged mammalian expression vector. The deletion mutants were generated by site-directed mutagenesis using DNA primers described in Supplementary Table S1. Mouse Traip gene (NM_011634.3) was amplified from cDNA pool of AB2.2 mouse ES cells, and then cloned into modified EF1a-myc-his plasmid (using two restriction enzyme sites, KpnI and NotI). Traip deletion mutants were generated by site-directed mutagenesis. Human NEIL3 gene (NM_018248.3) was amplified from cDNA pool of HeLa cells, and then cloned into EGFP-N1 mammalian expression vector. All constructs were confirmed by sequencing. Guide RNA plasmids for mouse Zfp212, mouse Traip, mouse Neil3, and mouse Fancd2, were generated by cloning guide sequences into pX330 (Addgene plasmid number 42230), respectively. The target sequences for gene editing were selected by using CRISPOR (www.crispor.tefor.net). The target sequences for each guide RNA were as follows and primer sequences for cloning are listed in the Supplementary Table S1:

Mouse Zpf212 guide #1: 5′-TGTGGCTGGAGCTAATGGCG-3′

Mouse Zfp212 guide #2: 5′-GGTGGCTGCTATTCAAGCCG-3′

Mouse Traip guide #1: 5′-CTTCGATCACTCCCGTGACG-3′

Mouse Traip guide #2: 5′-TGGCTCGACTCAAGCAGCCC-3′

Mouse Neil3 guide #1: 5′-ATCTTGATTAACCCACGGGA-3′

Mouse Neil3 guide#2: 5′-TCTGGTGAGCTGCACCGCCA-3′

Mouse Fancd2 guide#1: 5′-TTCCGCCATGATGCCGGCCA-3′

Mouse Fancd2 guide2#2: 5′-ACGACATGCACCTGGTGATC-3′

### Generation of knockout (KO) mouse ES cells (mESCs) for zfp212, traip, neil3 and fancd2 and stable mouse ES cells expressing mouse traip cDNAs

For Zfp212 KO, mouse AB2.2 and Fancb KO ES cells in Dulbecco's phosphate-buffered saline (DPBS) were electroporated with two guide RNAs (pX330, Addgene plasmid number 42230) which target sequences in exon 2 of mouse *Zfp212* (Zfp212 Guide RNA #1 and #2, Supplementary Figure S7A) coupled with *miniHPRT* expression plasmid. The condition for electroporation was as follows: 230 V, 500 μF (Bio-Rad Gene Pulser). Next, 250 μl of electroporation mixture was seeded onto a 10-cm gelatin-coated plate. The following day, final concentrations of 0.5× HAT (0.5 mM sodium hypoxanthine, 2 μM aminopterin, 80 μM thymidine, Gibco) were added. After 8–10 days in HAT, resistant colonies were picked, expanded, and then screened by western blotting for deletion of Zfp212 expression. For Traip KO, mouse AB2.2, Zfp212 and Fancb KO ES cells were electroporated with two guide RNAs which target sequences in the first ATG exon of mouse Traip (Traip gRNA #1 and #2, Supplementary Figure S6A) coupled with puro expression plasmid. The cells were seeded onto a 10-cm gelatin-coated plate. The next day, the cells were added and selected in 3 μg/ml of puromycin (Gibco) for 8–10 days. The resistant colonies were picked, expanded, and then screened by western blotting for deletion of Traip expression. For Neil3 KO, mouse AB2.2, Zfp212, Traip and Fancb KO ES cells were electroporated with two guide RNAs which target sequences in the third exon of mouse *Neil3* (Neil3 gRNA #1 and #2, Supplementary Figure S8A) coupled with hygromycin (Gibco) expression plasmid. The cells were seeded onto a 10-cm gelatin-coated plate. The next day, the cells were selected in 180 μg/ml of hygromycin for 8 to 10 days. The resistant colonies were picked, expanded, and then screened by genomic PCR and sequencing for deletion of Neil3 expression due to lack of antibody specific to mouse Neil3. For Fancd2 KO, mouse AB2.2 and Zfp212 KO ES cells were electroporated with two guide RNAs targeting sequences in the exon 18 of mouse *Fancd2* (Fancd2 gRNA #1 and #2, Supplementary Figure S8B) coupled with hygromycin expression plasmid. The cells were seeded onto a 10-cm gelatin-coated plate. The next day, the cells were selected in 180 μg/ml of hygromycin for 8–10 days. The resistant colonies were picked, expanded, and then screened by western blotting. All the positive clones for *each* KO were also sequenced to validate the edited alleles (Supplementary Table S3). DNA primers (test DF and test DR for each target gene) used for PCR and cloning to validate each edited allele were obtained from Macrogen and are listed in the Supplementary Table S1. For generation of mESCs stably expressing mouse Traip (mTraip) cDNAs, Traip KO mESCs were electroporated with either an empty vector or each mTraip cDNA-Myc-His vectors, and then seeded onto a 10-cm gelatin-coated plate. 36 hr post-electroporation, cells were selected in 8 μg/ml of blasticidin (Gibco) for 8–10 days. The resistant colonies were picked, expanded, and then screened by western blotting for Traip-Myc-His expression.

### Yeast two-hybrid screening

The cDNA of TRAIP was subcloned into pGBKT7 as the bait. The yeast two-hybrid screening was followed manufacture's instruction (Clontech). A HeLa cell cDNA library in pACT2 was used as the prey to screen ≈2 × 10^6^ colonies. The bait and the library DNAs were co-transformed into AH109 yeast strain using the lithium acetate method. The transformants were selected for growth on the Leu^−^, Trp^−^, His^−^ and Ade^−^ solid media containing 30 mM 3-aminotriazole (3-AT). The β-galactosidase assay was performed by incubating freeze-fractured colonies on nitrocellulose in Z-buffer (60 mM Na_2_HPO_4_, 40 mM NaH_2_PO_4_, 10 mM KCl, 1 mM MgSO_4_, 0.03 mM β-mercaptoethanol and 2.5 M X-gal) at 30°C for 30 min.

### Small interfering RNAs (siRNAs)

A list of all siRNA duplexes used in this work can be found in the Supplementary Table S1. Control and TRAIP siRNA were previously described (34,35). The sequences of ZNF212 siRNA used in this work are listed in the Supplementary Table S1. siRNAs (20 nM) for TRAIP, ZNF212 (GENOLUTION), and NEIL3 (Bioneer) were transfected into cells using Lipofectamine RNAiMAX reagent (Invitrogen, Carlsbad, CA) as indicated. Experiments were carried out 48 h after transfection.

### Antibodies

Anti-TRAIP, -GFP and -γH2AX antibodies were previously described (34,35). Other antibodies were purchased as the followings: anti-Flag-HRP (Sigma, A8592), anti-Myc-HRP antibody (Roche, 11814150001), anti-GFP (Clontech, 632380), anti-β-actin antibody (Sigma, A5441), anti-α-tubulin antibody (Millopore, 05–829), anti-ZNF212 antibody (Altas antibodies, HPA049807), anti-TRAIP antibody (30), anti-RAD51 antibody (Abcam, ab3801), anti-RPA2 antibody (Bethyl laboratories, A300-244A), anti-53BP1 (Cell signaling technology, #4937), anti-BRCA1 antibody (Santa cruz, SC-6954), anti-FANCD2 antibody (Novus Biologicals, NB100-182), anti-Histone 3 antibody (Santa cruz, sc-8654), anti-NEIL3 antibody (Proteintch, 11621-1-AP) and anti-MCM7 antibody (Santa cruz, sc-9966).

### hNEIL3 and hZNF212 purification

Human NEIL3 (hNEIL3) and ZNF212 (hZNF212) purification protocol was modified from the previous protocol (36). The hNEIL3 gene was subcloned into a pET19b-derived plasmid containing 3x FLAG at the N-terminus and 10x His at the C-terminus. 3X-Flag tagged coding region of human ZNF212 was cloned in pCold^TM^ TF DNA (3365, Takara). The hNEIL3 protein was expressed in *Escherichia coli* BL21 (DE3) strain (CP110, Enzynomics). For the purification of hNEIL3 and hZNF212, cells were grown at 37°C and 15°C, respectively in 4 L LB broth supplemented with 0.1 mM ZnSO_4_. When OD_600_ became ∼0.6, the protein was expressed with 1 mM IPTG (Isopropyl β-d-1thiogalactopyranoside) and further incubated at 16°C for 20 h. Cells were harvested at 4000 g for 20 min and resuspended in resuspension buffer (50 mM HEPES [7.5], 200 mM NaCl, and 0.01% NP-40) with 1x protease inhibitor (Halt, 78439, Thermo Fisher Scientific) and 1 mM PMSF (phenylmethylsulfonyl fluoride). All the following processes were performed at 4°C. The cells were lysed by sonication and successively ultracentrifuged at 60000 g for 40 min. The clarified lysate was filtered using 0.22 μm syringe filters (J1.F204.030N, Biofil) and then loaded into 10 ml of Ni-NTA gravity-flow column (HisPur™ Ni-NTA resin, 88222, Thermo Fisher Scientific), which was pre-equilibrated with Ni-wash buffer (50 mM HEPES [7.5], 200 mM NaCl, 0.1% NP-40, 5 mM Imidazole and 1 mM PMSF). Ni-NTA column was washed with 5× bed volume of Ni-wash buffer. Proteins were eluted with Ni-elution buffer (50 mM HEPES [7.5], 200 mM NaCl, 0.1% NP-40, 500 mM imidazole and 1 mM PMSF). Eluates were analyzed by 10% SDS PAGE. Fractions containing hNEIL3 proteins were pooled and diluted with 50 mM HEPES [7.5] and 0.1% NP-40 to reduce NaCl concentration from 200 to 100 mM. Next, hNEIL3 was purified through Q column (HiTrap Q HP 1 ml, 17115301, Cytiva) by salt gradient from 100 mM to 1 M in 50 mM HEPES [7.5], 1 mM DTT, 0.1% NP-40 and 1 mM PMSF. Fractions containing hNEIL3 were pooled and concentrated using 10K Amicon filters (UFC801024, Merck Millipore). The sample was then loaded into gel filtration column (Superdex 200 10/300 GL, Cytiva) in 50 mM HEPES [7.5], 200 mM NaCl, 1 mM DTT and 0.1% NP-40. Fractions containing hNEIL3 were pooled, and glycerol and DTT were added up to 50% and 1 mM, respectively. Concentration of hNEIL3 and hZNF212 was measured by BSA titration in 10% SDS PAGE.

### Transfection and immunoprecipitation

Transient transfection was performed by using poly(ethylenimine) (PEI, polysciences). For immunoprecipitation, cells were washed with ice-cold phosphate buffered saline (PBS), and then lysed in NETN buffer (0.5% Nonidet P-40, 20 mM Tris [pH 8.0], 50 mM NaCl, 50 mM NaF, 100 μM Na3VO4, 1 mM dithiothreitol (DTT) and 50 μg/ml phenylmethylsulfonyl fluoride (PMSF)) with benzonase (Enzynomics, M018H) at 4°C for 40 min. Crude lysates were cleared by centrifugation at 14000 rpm at 4°C for 5 min, and supernatants were incubated with protein A-agarose-conjugated primary antibodies, FLAG-M2 affinity gel (Sigma, Cat#A2220) or c-Myc Agarose affinity gel (Sigma, Cat#7470). The immunocomplexes were washed three times with NETN buffer, and then subjected to sodium dodecyl sulfate-polyacrylamide gel electrophoresis (SDS-PAGE). Western blotting was performed using the antibodies indicated in the figure legend. Proteins were visualized using secondary horseradish peroxidase-conjugated antibodies (Enzo Life Sciences, New York, NY) and enhanced chemiluminescence reagent (Thermo Fisher Scientific). Signal was detected using an automated imaging system (ChemiDoc™; Bio-Rad Laboratories, Hercules, CA).

### Laser microirradiation and imaging of cells

An accumulation of GFP-fused ZNF212 wild type and mutants, mCherry-fused ZNF212 wild type, GFP-fused TRAIP wild type and GFP-fused NEIL3 was analyzed as described previously (37). For the condition of angelicin and trioxsalen (TMP), microirradiation was performed 1h post- treatment with either angelicin (20 μM) or TMP (20 μM). Single strand breaks or DSBs of DNA were induced using a LSM 880 laser microirradiation system (Carl Zeiss). HeLa and U2OS cells in 35mm confocal plates were transfected with indicated GFP-tagged expression vector for 24 h, and then were incubated with 10 μM of 5-brome-2′-deoxyuridine for 20 h before laser-induced DSBs. Ten cells per well were subjected to laser-induced DSBs during 10 s using the ×40 water objective. Fixed wavelength of ultraviolet A laser (355 nm) was used for laser microirradiation in the temperature-controlled chamber with CO_2_ supplier. After laser treatment, cells were incubated at 37°C for the indicated times. The intensity of each laser stripe in each time point was determined using confocal microscope. The kinetic analyses were performed using the ZEN (blue edition) software from ZEISS Microscopy. For immunostaining, cells were fixed with 3% paraformaldehyde for 10 min, and then permeabilized with 0.5% Triton X-100 in PBS for 5 min at room temperature. Samples were blocked with 5% goat serum and then incubated with the primary antibody for 1 h. The fixed cells were incubated with the indicated antibodies diluted in PBS supplemented with 10% fetal bovine serum at 4°C overnight. After three washes with 0.05% Triton X-100 in PBS, Fluorescent-conjugated secondary antibodies (Thermo Fisher Scientific) were added and incubated for 30 min. Cells were mounted using ProLong® Gold antifade reagent (Vector Laboratories). Confocal images were acquired with an LSM880 confocal microscope (Carl Zeiss). Image acquisition and analysis were performed with the ZEN2.1 software.

### Cell survival assay

Cell survival assay was performed as previously described (34,35). HeLa cells in a 30 mm-diameter plate were transfected twice with the indicated siRNAs at 24-h intervals. Forty-eight hours after the second transfection, transfected cells were counted, seeded at indicated cell number, and then treated with DNA damaging agents at the indicated doses in the figure of cell survival. Seven days after treating with DNA damaging agents, cells were washed with PBS, fixed, stained with 2% (w/v) methylene blue, and the colonies were counted. The dose response/cell survival of mESCs for hydroxyurea (HU), mitomycin C (MMC) and camptothecin (CPT) was performed as previously described (33). Briefly, 2000 cells were seeded onto wells of a 24-well plate (day 0). On day 1, the media was replaced with each DNA damage agent at the doses shown in each figure. Cells were counted on day 6 using a hemacytometer. Two to three clones of KO mES cells were compared to the parental ES cells, AB2.2. These experiments were repeated three times. The dose response to trioxsalen was carried out according to the protocol previously described (17) with minor modifications. Two thousand cells were seeded onto wells of a 24-well plate (day 0). On day 1 the media was changed with trioxsalen (Sigma-Aldrich) at the doses shown in Figure 5, and then cultured for 1 hr. Cells were then exposed to 6 kJ m^−2^ of UVA light (365 nm, VL-6.L lamp) to photoactivate trioxsalen. The cells were washed twice with culture media, incubated at 37°C for 10 min to remove unbound trioxsalen, then washed again and treated with 12 kJ m^−2^ UVA to convert trioxsalen monoadducts into ICLs. Cells were then counted on day 6 using a hemacytometer.

### Homologous recombination assay

Homologous recombination assay was performed as previously described (38). U2OS DR-GFP cells were first treated with the indicated siRNAs in 24-well plates, and then transfected with 0.4 μg of I-SceI and indicated plasmids per well 24 h after siRNA transfection. Seventy-two hours after I-SceI transfection, cells were trypsinized, and then the percentage of GFP positive cells was determined by flow cytometry. BD FACSCanto II (NFEC-2011-02-145106) is used for FACS analysis at the Core Facility Center for Chronic and Metabolic Diseases at Sookmyung Women's University.

### Analysis of metaphase chromosomes and sister chromatin exchange (SCE)

For chromosome analysis, mouse ES cells were incubated for 4 h with 1 μg/ml colcemid, and then metaphase cells were harvested by trypsinization. The cells were incubated in 75 mM KCl for 15 min at 37°C, and then fixed with methanol:acetic acid (3:1) twice. Cells were dropped onto glass microscope slides, aged and then stained with 5% Giemsa stain. Images were acquired using a fluorescence microscope (BX53; Olympus). Thirty-five metaphase cells were taken randomly at the indicated condition for a statistic analysis (unpaired t test). For the SCE assay, HeLa cells and mouse ES cells were cultured in the presence of 25 μg/ml of 5-bromo-2’-deoxyuridine (BrdU) for 48 and 21 h, respectively. After BrdU treatment, cells were treated for 3–4 h with 0.2 μg/ml of colcemid, and then harvested by trypsinization. The cells were then processed as described in the section of chromosome analysis. The prepared slides were treated with 1 μg/ml of Hoechst 33258 (Invitrogen) for 30 min, and then exposed to 265 nm of UV light for 30 min. Slides were incubated 15 min in 2× SSC at 65°C, and then stained with 5% Giemsa solution. Images were acquired using a fluorescence microscope (BX53; Olympus). Thirty-five metaphase cells were taken randomly from each condition for analysis (unpaired t test).

### iPOND assay

The iPOND assay was performed as previously described (39) with minor modifications. mESCs were first treated with 200 nM of MMC, and then 20 μM of EdU was added 30 min after MMC addition, and then incubated for 3.5 h under MMC treatment. Cells were subsequently fixed using 1% of formaldehyde for 20 min at RT. The crosslinking reaction was quenched using 0.125 M glycine, and then the cells were washed three times with PBS. Cells were incubated with 0.25% of Triton X-100™ in PBS for 30 min at RT, and then were pelleted. Permeabilization was stopped with 0.5% bovine serum albumin in PBS. Cells were pelleted again and washed with PBS. After centrifugation, cells were resuspended with a click reaction solution and incubated for 1 h at RT on a rotator. After centrifugation, the click reaction was stopped by resuspending cells in PBS containing 0.5% of bovine serum albumin (BSA). Cells were then pelleted and washed with PBS twice. Cells were resuspended in lysis buffer and sonicated. Lysates were cleared and then incubated with streptavidin-agarose beads overnight at 4°C in the dark. The beads were washed once with lysis buffer, once with 1 M NaCl, and then twice with lysis buffer. To elute proteins bound to nascent DNA, the 2× sodium dodecyl sulfate Laemmli sample buffer was added to packed beads (1:1; v/v). Samples were incubated at 95°C for 30 min, followed by immunoblotting with the indicated antibodies

## RESULTS

### ZNF212 is associated with TRAIP

**Figure 1.**
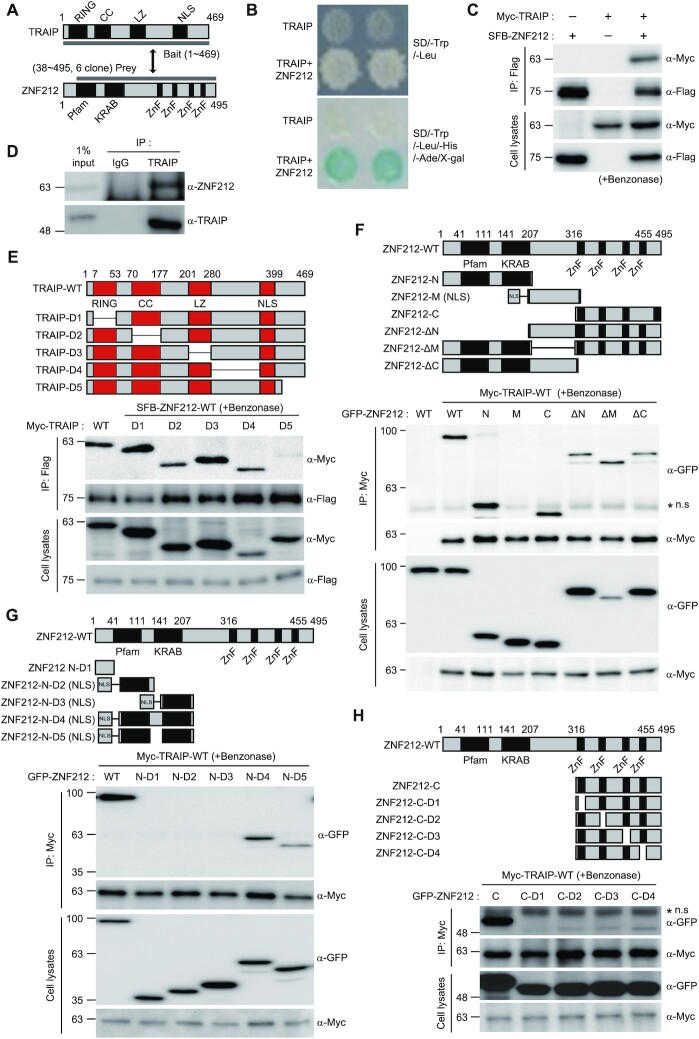
ZNF212 is associated with TRAIP. (**A**) Schematic illustration of yeast two-hybrid screening with the full-length TRAIP as bait. (**B**) TRAIP and ZNF212 were co-transformed into yeast AH109 cells and growing colonies were assessed under the stringent nutritional selection. Blue colonies on the selective plate indicate a positive interaction. (**C**) The interaction between exogenous Myc-TRAIP and SFB-ZNF212. The indicated plasmids were transfected into 293T cells. 48 h post-transfection, transfected cell lysates with benzonase were immunoprecipitated (IP) using anti-Flag bead and subjected to Western blotting analysis using the indicated antibodies. (**D**) The interaction between endogenous TRAIP and ZNF212. IP reaction with benzonase was performed using rabbit IgG or anti-TRAIP antibodies, and then subjected to Western blot analysis using the indicated antibodies. (**E**) The mapping of the domain of TRAIP responsible for ZNF212 interaction. Upper panel shows diagram of wild type (WT) TRAIP and serial deletion mutants (D1 to D5). Numbers indicate amino acids. SFB-ZNF212 and Myc-TRAIP WT and mutants were co-transfected into 293T Cells. Cell lysates with benzonase were immunoprecipitated with Flag-bead. The interaction with ZNF212 was detected with anti-Myc antibody. (**F**) The mapping of the domain of ZNF212 responsible for TRAIP interaction. Upper panel shows diagram of ZNF212 WT and deletion mutants (N, M, C, ΔN, ΔM and ΔC). Numbers indicate amino acids. 293T cells were co-transfected with plasmids encoding Myc-TRAIP and either GFP-ZNF212 WT or deletion mutants. 24 h post-transfection, cell lysates with benzonase were subjected to IP with anti-Myc bead, and then immunoblotted with the indicated antibodies. Myc-TRAIP, GFP-ZNF212 WT and deletion mutants in the lysates were analyzed by immunoblotting and shown in the bottom panel. Asterisk indicates the crossreacting band. (**G**) The mapping of the domain in ZNF212 N responsible for TRAIP interaction. Upper panel represents the diagram of WT, ZNF212 N and NLS-domain fragments and deletion mutant (ZNF212 N-D1 and NLS-N-D2 to NLS-N-D5). (**H**) The mapping of the domain in ZNF212 C responsible for TRAIP interaction. Upper panel represents the diagram of WT, ZNF212 C and deletion mutants (ZNF212 C-D1 to C-D4). The interaction between TRAIP and either ZNF212 deletion mutants or NLS-fragments was tested as described in (F).

TRAIP functions in DDR, HR and ICL repair pathways either via E3 ligase activity and through interactions with other proteins, including PCNA, RNF20/40 and RAP80 (17,27–31). The TRAIP-PCNA association is important for counteracting with replication stress (27), while the TRAIP-RAP80 and TRAIP-RNF20/40 interactions are needed for DDR and HR (30). As an E3 ligase, TRAIP plays a master role in NEIL3 and FA dependent ICL repair pathways (17,32). To better understand molecular basis of TRAIP activity, we set out to identify novel effectors or substrates using a yeast two-hybrid screen with the full-length TRAIP as a bait and a human HeLa cDNA library as a prey (Figure 1A). Among 2 × 10^6^ transformants, 90 positive clones with the highest galactosidase activity were obtained (Supplementary Table S2). Sequence analysis revealed that the Zinc finger protein 212 (ZNF212), a gene of unknown function, was recovered with the highest number of hits, six clones (Figure 1A and Supplementary Table S2): One for the N-terminal region, one for the KRAB motif and four for the C2H2 Zinc finger (ZF) motifs in the C-terminus. Proteins with the zinc finger motifs have been shown to play roles in DDR and DNA repair (40). Thus, we decided to test possible roles of ZNF212 in DDR and DNA repair. First, we confirmed the potential interaction between TRAIP and ZNF212 by targeted yeast two-hybrid analysis (Figure 1B) and co-immunoprecipitation (IP) in cells overexpressing SFB-ZNF212 and Myc-TRAIP (Figure 1C). The co-immunoprecipitation and Western blot data hereafter are representative from at least three independent experiments. Using a ZNF212 specific antibody, we were able to detect endogenous ZNF212 in the endogenous TRAIP immunoprecipitates (Figure 1D). To identify the responsible domains for ZNF212 and TRAIP interaction, we generated a series of internal deletion mutants of Myc-TRAIP (TRAIP-D1 to -D5) and transfected the DNA constructs individually into HEK293T cells expressing SFB-ZNF212 wild type (SFB-ZNF212-WT). To exclude that the interaction between ZNF212 and TRAIP is mediated by nucleic acids, we performed immunoprecipitation assay in the presence of the Benzonase. By immunoprecipitation and immunoblotting analysis, we found that the Myc-TRAIP-D1 to -D4 associated with SFP-ZNF212-WT, but Myc-TRAIP-D5 failed to do so, demonstrating that C-terminus of TRAIP is responsible for the interaction (Figure 1E). In the reciprocal experiment, we generated a series of ZNF212 deletion mutants and performed immunoprecipitation assays to determine responsible elements for TRAIP interaction (Supplementary Figure S1, upper panel). Unexpectedly, we found that all of ZNF212 deletion mutants were associated with Myc-TRAIP (Supplementary Figure S1). Thus, we generated additional GFP-tagged ZNF212 deletion mutants, such as ZNF212-N, -M, and -C, and ZNF212-ΔN, -ΔM and -ΔC (Figure 1F, upper panel), and performed immunoprecipitation assay. All deletion mutants, except ZNF212-M, associated with Myc-TRAIP, suggesting that either ZNF212-N or -C terminus is sufficient for the interaction (Figure 1F). To narrow down the TRAIP binding region of ZNF212, the N-terminus was divided into five regions, ZNF212-N-D1 to -D5 (Figure 1G) and the individual ZF motif in the C-terminal domain was also removed to generate deletion mutants, ZNF212-C-D1 to -D4 (Figure 1H). Co-immunoprecipitation analysis using these deletion mutants revealed that both amino acid residues 41–207 and each of the ZF motifs in C-terminus are responsible for the interaction with TRAIP (Figure 1G and H). Taken together, these findings demonstrate that the ZNF212 is a bona fide novel interaction partner of TRAIP.

### The interaction between TRAIP and ZNF212 promotes the recruitment of both proteins to sites of DNA damage

**Figure 2.**
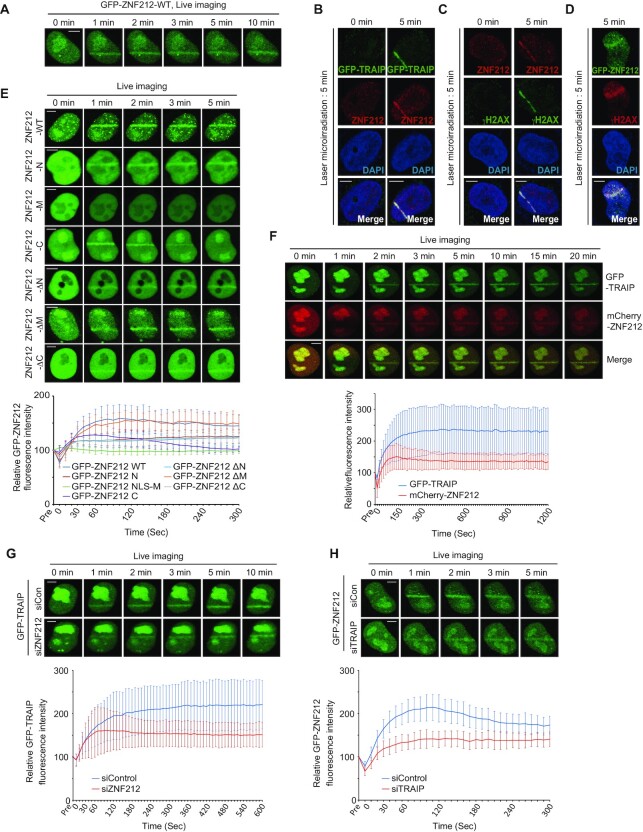
ZNF212 is localized to the DNA damage sites through its interaction with TRAIP. (**A**) HeLa cells were transfected with GFP-ZNF212, and then treated with laser microirradiation 24 h post-transfection in the presence of BrdU. We examined the laser strip at each indicated time. (**B**) HeLa cells were transfected with GFP-TRAIP, and then treated with laser microirradiation 24 h post-transfection in the presence of BrdU. 5 min after microirradiation, the cells were fixed and stained with anti-ZNF212 antibody (red). 4′,6-Diamidino-2-phenylindole (DAPI) was used to stain the nucleus. (**C**) HeLa cells were treated with laser microirradiation in the presence of BrdU. 5 min after microirradiation, the cells were fixed and stained with anti-ZNF212 antibody (red) and anti-γH2AX antibody (green), a marker for DNA damages. (**D**) HeLa cells were transfected with GFP-ZNF212, and then treated with laser microirradiation 24 h post-transfection in the presence of BrdU. 5 min post-irradiation, the cells were fixed, and then stained with anti-γH2AX antibody (red). (**E**) ZNF212 translocation to DNA damage sites via its N and C-terminal regions, both. HeLa cells were transfected with GFP-ZNF212 WT or deletion mutant plasmids, and then treated with laser microirradiation 24 h post-transfection in the presence of BrdU. (**F**) The recruitment kinetics of GFP-TRAIP and mCherry-ZNF212 translocation to DNA damage sites. (**G**) HeLa cells treated with either control or ZNF212 siRNAs were transfected with GFP-TRAIP, and then treated with laser microirradiation 24 h post-transfection in the presence of BrdU. (H) HeLa cells treated with either control or TRAIP siRNA were transfected with GFP-ZNF212, and then treated with laser microirradiation 24 h post-transfection in the presence of BrdU. The initial intensity of region of interest (ROI) before bleaching was calculated as 100% in each cell, and then the average intensity of the laser stripes was plotted. For each experiment, ten cells were analyzed. Data represent the mean ± SD from three independent experiments. Scale bar, 5 μm

It has been reported that TRAIP localizes to sites of DNA damage (28,30). As ZNF212 was identified as a TRAIP binding protein, we tested if ZNF212 also moves to sites of DNA damage. To this end, a laser microirradiation assay using UVA laser (355 nm) was employed in HeLa cells expressing GFP-ZNF212. As shown in the Figure 2A, live cell imaging analysis showed that GFP-ZNF212 accumulated at the laser stripes with a peak at approximately 3 minutes and remained there at later time points (Figure 2A). Using the ZNF212 specific antibody, we confirmed that endogenous ZNF212 accumulated at the laser-induced DNA lesions together with GFP-TRAIP (Figure 2B). Both endogenous ZNF212 and overexpressed GFP-ZNF212 were found at the DNA lesions marked with γH2AX (Figure 2C and D). Next, to identify domains of ZNF212 responsible for the translocation to the sites of DNA damage, we transiently overexpressed GFP-tagged ZNF212 deletion mutants in HeLa cells, and then measured the relative intensity of each deletion mutants in which GFP signals were accumulated at the sites of DNA damage. As shown in the Figure 2E, wild type ZNF212 (ZNF212-WT), ZNF212-N and ZNF212-C accumulated on the laser stripes although the fluorescent intensity of ZNF212-N and ZNF212-C were relatively lower compared to that of the wild type. On the other hand, we found that GFP-ZNF212-ΔM showed a similar level of recruitment to that of GFP-ZNF212-WT, suggesting that both N- and C-terminus of ZNF212, but not the central region have a role in the localization of ZNF212 to sites of DNA damage. Consistently, GFP-ZNF212-ΔN and GFP-ZNF212-ΔC, which contain either C- or N-terminus of ZNF212, respectively, are accumulated on the laser stripes with reduced intensity (Figure 2E). In order to narrow down specific regions of ZNF212 responsible for translocation to the DNA damage sites, we generated an additional four deletion mutants for the C-terminus and six for the N-terminus (Supplementary Figure S2A and B). By performing microirradiation assays with cells expressing the individual mutants, we found that four ZFs at C-terminus and amino acid residues 1–140 at N-terminus were important for translocation of ZNF212 to the sites of DNA damage (Supplementary Figure S2A and B). Overall ZNF212 domains required for ZNF212 localization to the laser stripes are also important for the interaction with TRAIP (Figures 1G and H). Taken together, our data imply that ZNF212 travels to the sites of DNA damage through the interaction with TRAIP. Interestingly, the amino acid residues 1–40 of ZNF212 were not associated with TRAIP, but localized to DNA damage sites, suggesting that the amino acid residues 1–40 of ZNF212 may interact with an unknown factor for the translocation.

To study the kinetics of the recruitment of TRAIP and ZNF212 to DNA lesions, we employed real-time imaging to monitor protein accumulation at laser stripes induced by microirradiation. In HeLa cells expressing GFP-TRAIP and mCherry-ZNF212, both proteins were recruited to the sites of DNA-damage within 1 min after laser microirradiation (Figure 2F). We observed that the accumulation of TRAIP-GFP and mCherry-ZNF212 peaked at approximately 3 min after irradiation, and that both proteins remained stably associated with damage at later time points (Figure 2F). These data suggest that ZNF212 acts together with TRAIP in response to DNA damage. Since TRAIP and ZNF212 interact with each other and co-localize to sites of DNA damage, we next asked whether they regulate each other's recruitment to DNA lesions. To test this, we analyzed the kinetics of accumulation of GFP-TRAIP or GFP-ZNF212 on laser stripes when either ZNF212 or TRAIP was knocked down. Interestingly, we found that accumulation of GFP-TRAIP was significantly reduced in ZNF212-depleted cells (Figure 2G) and similarly localization of GFP-ZNF212 (Figure 2H) was severely reduced in TRAIP-depleted cells. To rule out the off-target effects of siRNA, we re-introduced siRNA-resistant mCherry-ZNF212 or mCherry-TRAIP together with corresponding siRNAs and found that led to a rescue of the accumulation defect (Supplementary Figure S2C-S2E). In addition, depletion of either TRAIP or ZNF212 had no impact on the expression levels of each other (Supplementary Figure S2F). Altogether, our data suggest that TRAIP and ZNF212 directly interact in a complex that localizes to the sites of DNA damage.

### ZNF212 is important for cell survival in response to replication-blocking DNA damage and for HR in collaboration with TRAIP

**Figure 3.**
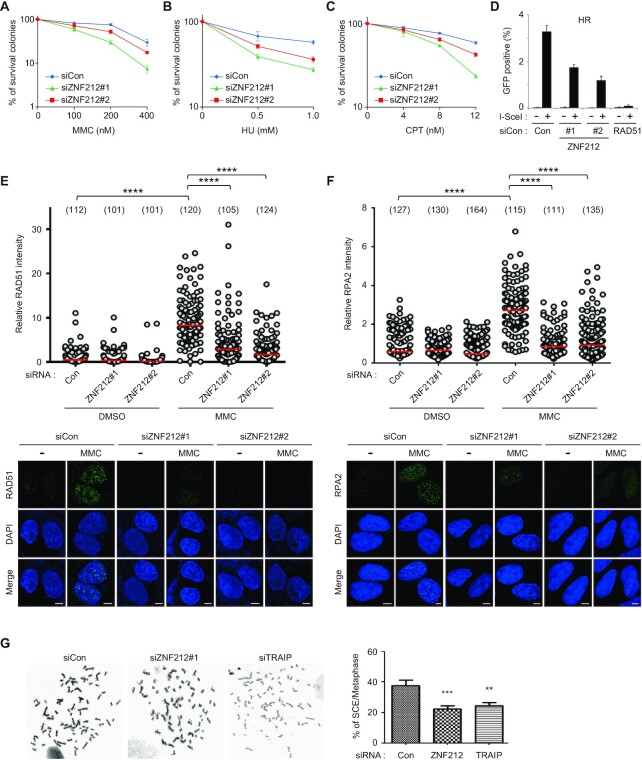
ZNF212 participates in DDR for cell survival in response to replication-associated damages and HR. (**A**–**C**) The cell viability of control or ZNF212 depleted HeLa cells after the treatment of various DNA damaging agents, including MMC, HU and CPT. Data represent the mean ± SEM from three independent experiments. (**D**) DR-GFP based HR assays in U2OS cells depleted for ZNF212. siRAD51 was used as positive control for HR reduction. The graph represents the mean ± SEM from two independent experiments DR-GFP based HR assays. (**E**, **F**) MMC-induced RAD51 or RPA2 foci formation in ZNF212 depleted U2OS cells. U2OS cells depleted with either control or ZNF212 siRNAs were exposed to 0 or 500 nM of MMC for 16 hr, and then fixed and subjected to staining with indicated antibodies. The intensity of RAD51 or RPA2 foci per nucleus was scored for each sample using the ZEN Blue software (Carl Zeiss). Statistical analysis was performed by unpaired t-test using Prism 8 software (GraphPad). ^****^P< 0.0001. (**G**) Depletion of either ZNF212 or TRAIP in HeLa cells reduced sister chromatid exchange (SCE), an indication of HR. Thirty-five metaphase cells were counted for the indicated condition. Data represent the mean ± SD. Unpaired *t* test was performed for statistics (Prism 8 software). **P< 0.01, ***P< 0.001.

Since TRAIP was shown to be important for cell survival in response to DNA replication inhibition (27,28,30), we examined whether ZNF212 has similar roles. We used two siRNAs #1 and #2 for ZNF212 depletion for all experiments, both of which showed the decent levels of ZNF212 knockdown (Supplementary Figure S3A). First, we tested the effects of ZNF212 depletion on clonogenic survival of HeLa cells in response to drugs that lead to replication-blocks, including MMC (DNA interstrand crosslinks), hydroxyurea (HU, dNTP depletion), and camptothecin (CPT, topoisomerase inhibitor). We found that depletion of ZNF212 leads to moderately enhanced sensitivity to MMC, HU and CPT compared to HeLa cells treated with control siRNA (Figure 3A–C and Supplementary Figure S3B–D). Next, we tested whether ZNF212 has a role in HR as we previously observed for TRAIP (30). Using a DR-GFP based HR reporter assay, we found that HR efficiency was decreased by about 50% in ZNF212-depleted cells (Figure 3D and Supplementary Figure S3E). These findings were further supported by the observations that MMC-induced RAD51 foci formation, a key indicator of HR proficiency, was severely impaired in ZNF212-depleted cells (Figure 3E). Similarly, damage-induced RPA2 foci formation was significant decreased in ZNF212-depleted cells (Figure 3F). Given that the expression levels of RAD51 and RPA2 was not altered in cells treated with siRNA against ZNF212 (Supplementary Figure S3F), these findings suggest that ZNF212 participates in DNA damage response. At the chromosome level, spontaneous sister chromatid exchange (SCE) has been known as an indication of HR. Therefore, we performed SCE assay in HeLa cells and found that depletion of ZNF212 resulted in significant decrease of SCE compared to cells treated with control siRNA (Figure 3G).

**Figure 4.**
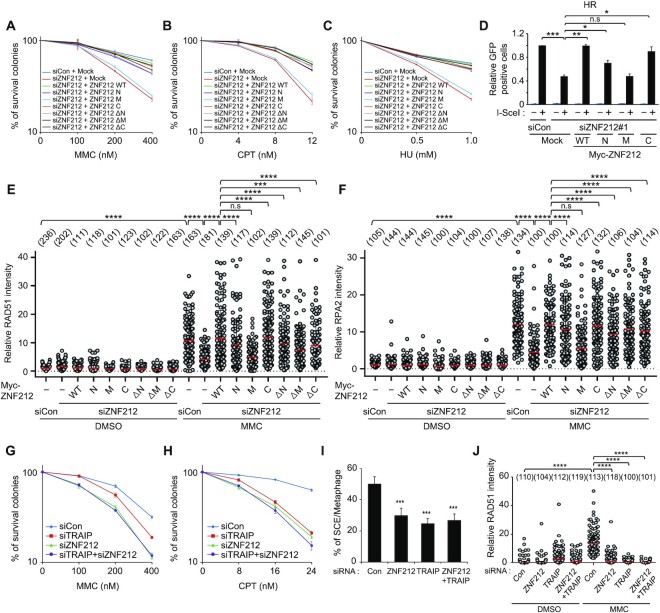
Expression of siRNA resistant ZNF212-N or -C rescues defects in DDR and DNA repair in cells depleted with endogenous ZNF212. (**A**–**C**) Clonogenic assay of ZNF212-depleted HeLa cells and the expression of each indicated plasmid after the treatment of various DNA damaging agents, including MMC, HU and CPT. HeLa cells depleted with either siControl or siZNF212 were transfected with each following plasmid (Myc-ZNF212 WT, N, M, C, ΔN, ΔM and ΔC), and then 200 or 400 cells were plated and treated with indicated doses of DNA damaging agents. The number of surviving colonies was counted 7 days after treatment of DNA damaging agents. Data represent the mean ± SEM from three independent experiments. (**D**) Expression of ZNF212WT, N- and C-terminal region restored the homologous recombination in DR-GFP based HR assays. The average of three independent experiments is shown. Error bars indicate standard deviation (SD). (**E**, **F**) MMC-induced RAD51 and RPA2 foci formation in ZNF212 depleted U2OS cells restored by reconstitution of ZNF212. U2OS cells depleted with either control or ZNF212 siRNAs were transfected with Myc-ZNF212 WT, N, M, C, ΔN, ΔM and ΔC, respectively. And the transfected cells were exposed to 0 or 500 nM of MMC for 16 hr, and then fixed and subjected to staining with indicated antibodies. The intensity of RAD51 or RPA2 foci per nucleus was scored for each sample using the ZEN Blue software (Carl Zeiss). (**G**, **H**) Comparison of sensitivity of HeLa cells to DNA replication-blocking agents, MMC and CPT. HeLa cells transfected with the indicated siRNA (*N* = 200 or *N* = 400) were plated and treated with indicated doses of each DNA damaging agent. The number of surviving colonies was counted 7 days after treatment of each DNA damaging agent. Data represent the mean ± SEM from three independent experiments. (**I**) Decreased SCE in HeLa cells transfected with indicated siRNAs. Thirty-five metaphase cells were counted at the indicated condition. Data represent the mean ± SD. (**J**) ZNF212 and TRAIP are epistatic for MMC-induced RAD51 foci formation. U2OS cells depleted with indicated siRNAs were exposed to 0 or 500 nM of MMC for 16 h, and then fixed and subjected to staining with indicated antibodies. The intensity of RAD51 foci per nucleus was scored for each sample using the ZEN Blue software (Carl Zeiss). Statistical analysis was performed by unpaired *t*-test using Prism 8 software (GraphPad). ^****^P< 0.0001.

To exclude the off-target effects of ZNF212 siRNAs, restoration experiments for cell survival, HR and damage-induced foci formation of RAD51 and RPA2 were conducted using the siRNA-resistant ZNF212 expression DNA constructs (Supplementary Figure S4A). The reconstitution with siRNA resistant Myc-tagged ZNF212-WT, -N, -C, -ΔN, -ΔM and -ΔC in cells depleted with endogenous ZNF212 successfully rescued the sensitivity to MMC, CPT and HU (Figure 4A–C and Supplementary Figure S4B-S4E), homologous recombination capacity (Figure 4D and Supplementary Figure S4F) and damage-induced RAD51 and RPA2 foci formation (Figure 4E, F and Supplementary Figure S4G–I). However, we found that Myc-ZNF212-M, which contains neither the N- nor C-terminus of ZNF212 failed to rescue all the defects, highlighting the importance of the interaction between ZNF212 and TRAIP for its activity. Taken together, our findings suggest that ZNF212 is important for cell survival and HR-dependent DNA repair in response to replication-blocking damage. Next, we asked whether ZNF212 is epistatic to TRAIP in response to DNA damaging agents. To test this, cells were treated with siRNA against ZNF212 or TRAIP individually and combined together and were subjected to MMC and CPT sensitivity assays, sister chromatin exchange assay and damage-induced RAD51 foci formation. As shown in the Figure 4G–J, we found that single and double knock-down for ZNF212 or TRAIP resulted in a similar level of MMC (Figure 4G and Supplementary Figure S5A) and CPT hypersensitivity (Figure 4H and Supplementary Figure S5B), efficiency of SCE (Figure 4I and Supplementary Figure S5C) and damage-induced RAD51 foci formation (Figure 4J and Supplementary Figure S5D). The expression level of RAD51, RPA2, TRAIP and ZNF212 was not altered in ZNF212- and TRAIP-depleted cells, respectively (Supplementary Figure S2F). These observations indicate that ZNF212 and TRAIP are indeed epistatic in the DNA damage response. Taken together, these data demonstrate that ZNF212, as a binding partner for TRAIP, plays an important role in DNA damage response and DNA repair pathways.

### ZNF212 functions upstream of neil3 and FA pathways in collaboration with TRAIP for ICL repair in mouse embryonic stem cells

In *Xenopus* egg extracts, TRAIP has been shown to regulate the choice between two replication-coupled ICL repair pathways for psoralen and abasic site ICLs: the NEIL3 and FA pathways (17). In human cells, it was reported that TRAIP is non-epistatic to both NEIL3 and FA pathways for psoralen-ICL repair (32). We found that ZNF212 interacts with TRAIP, and ZNF212 deficiency led to sensitivity to MMC, so we hypothesized that ZNF212 might also participate in this pathway choice. To address the functional roles of ZNF212 and genetic interaction between ZNF212 and TRAIP in the two pathways, we employed mouse embryonic stem cells (mESC, AB2.2), which is a highly replicating cell line and therefore dependent on replication-coupled repair pathways. We genetically deleted the mouse *Traip* gene in AB2.2 using CRISPR-Cas9 mediated gene editing (Supplementary Figure S6A and B). We verified that mESCs deleted for *Traip* show the expected phenotypes (27,28,30), including cell cycle arrest at G2 phase and hypersensitivity to MMC (Supplementary Figure S6C–F). To define specific mouse Traip domains responsible for the Traip functions in genome maintenance, we established Traip KO mESC lines stably expressing wild type Traip and a series of Traip deletion mutants (Supplementary Figure S6G–I). Expression of either Ring domain, mTraip-D1, or mTraip-R18C mutant, a hypomorphic TRAIP variant (17,29), failed to restore resistance to HU or MMC, showing that an intact E3 ubiquitin ligase activity is required for counteracting replication stress and repairing ICL damage (Supplementary Figure S6J and K). Expression of each interaction-defective mutant for either Zfp212 (mTraip-D5 and mTraip-D5 + PIP) or PCNA (mTraip-ΔPIP) failed to restore resistance to MMC or HU with different levels of sensitivity, implying these interactions are required to counteract the deleterious effects of MMC and HU (Supplementary Figure S6L and M). We also generated knockout mESCs of Zfp212 (Zfp212 KO), the mouse homolog of human ZNF212 (Supplementary Figure S7A and B). We found that Zfp212 KO mESCs were sensitive to MMC and CPT and showed decreased SCE capacity (Supplementary Figure S7C–E), as we observed in human cells. In addition, Zfp212 is epistatic to Traip for repairing MMC or CPT induced DNA damage (Supplementary Figure S7F–H). As shown in the HeLa cells previously (32), deletion of Traip or Zfp212 in mESC showed no discernible impact on damage induced monoubiquitination of Fancd2 (Supplementary Figure S7I and S7J). Taken together, our data indicate that mouse AB2.2 cells recapitulate TRAIP and ZNF212 functions in human cells, and we used the AB2.2 cells for understanding molecular basis of ZNF212 in ICL repair pathways.

**Figure 5.**
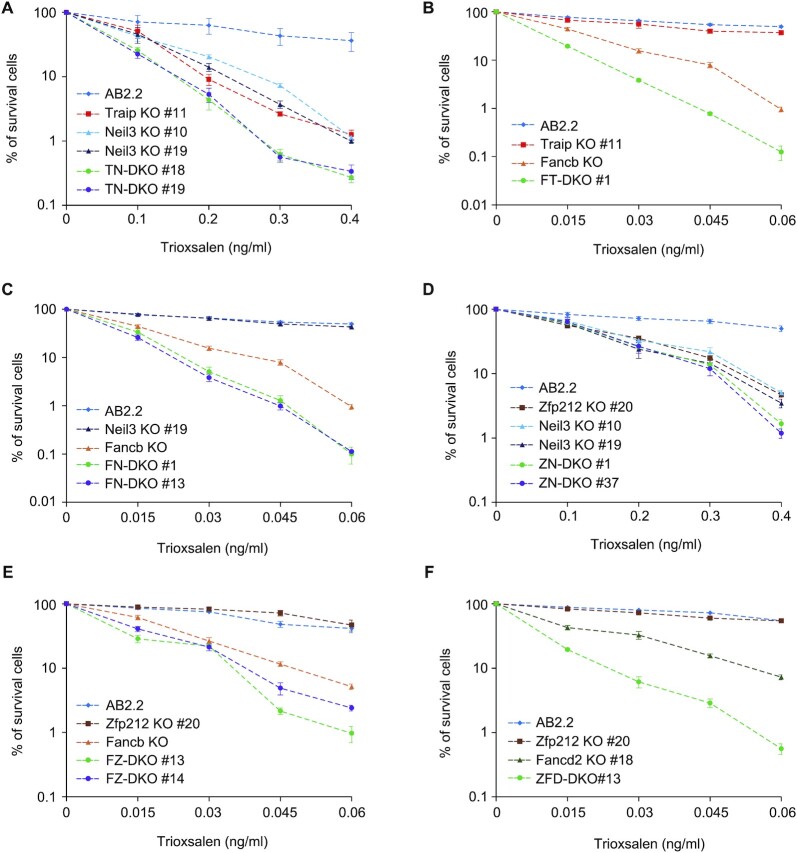
Systematic epistatic analysis of TMP sensitivity using gene knockout mESC lines. Dose–response curve to trioxsalen (TMP) in indicated mESCs (**A**–**F**). DKO indicates a double knockout. Data represent the mean ± SEM from three independent experiments.

Next, we asked how Zfp212 and Traip affect ICL repair by either the Neil3 or FA pathways in mESCs. To address this, we generated a set of additional gene knockout mESCs lines: Neil3 KO, Fancb KO (41), Fancd2 KO, Zfp212 and Traip double knockout (ZT-DKO), Zfp212 and Neil3 DKO (ZN-DKO), Traip and Neil3 DKO (TN-DKO), Fancb and Zfp212 DKO (FZ-DKO), Fancb and Traip DKO (FT-DKO), Fancb and Neil3 DKO (FN-DKO), Zfp212 and Fancd2 DKO (ZFD-DKO). These cells were generated using the paired gRNAs for each target gene coupled with Cas9 as described (Supplementary Figure S8 and the method section of generation of knockout mouse ES cells for Zfp212, Traip, Neil3 and Fancd2). We used trioxsalen (TMP), a psoralen derivative, to evaluate ICL repair capacity as TMP produces up to 90% of ICLs following UVA treatment. TMP-induced ICLs can be repaired by both the NEIL3 and FA pathways (17). Since Fancb KO mESCs exhibited extreme hypersensitivity to ICL forming agents compared to Traip KO, Zfp212 KO, and Neil3 KO mESCs, it was necessary to adjust the TMP dose range depending on the cell line used. We tested genetic interactions of TMP sensitivity with the set of knockout mESCs in the following dose ranges: 0.015–0.06 ng/ml for Fancb KO group and 0.1–0.4 ng/ml for Neil3 KO group. We found that deletion of *Traip* gene or *Neil3* gene resulted in hypersensitivity to TMP induced DNA damage (Figure 5A). In addition, consistent with the previous studies (17,32), we noticed that Traip and Neil3 double knockout mESCs (TN-DKO) showed enhanced sensitivity to TMP compared to individual Neil3 or Traip knockout mESCs, demonstrating that Traip is non-epistatic to Neil3 in mouse embryonic stem cells (Figure 5A). Similarly, we found that deletion of *Traip* gene in Fancb mESCs increased sensitivity to TMP compared to Fancb KO mESCs (Figure 5B), showing that Traip is non-epistatic to Fancb, a critical factor for the FA pathway. In addition, as shown in the Figure 5C, deletion of *Neil3* gene in Fancb KO mESCs led to increased sensitivity to TMP-ICL. Given the importance of Fancb in the FA pathway, this result suggests that Neil3 and FA pathways are non-epistatic in TMP-induced ICL repair in mESCs (Figure 5C). To understand genetic interaction between Zfp212 with Neil3 or Fancb in the TMP-ICL repair pathway, we performed sensitivity assay with TMP treatment. We found that Zfp212 KO mESCs showed hypersensitivity to TMP-ICL, which was further exacerbated by deletion of *Neil3* gene or *Fancb* gene, suggesting that Zfp212 is non-epistatic to Neil3 and Fancb (Figure 5D and E). To confirm the genetic interaction between Zfp212 and a second component of the FA pathway, we deleted *Zfp212* gene in Fancd2 KO mESCs and tested resistance to TMP treatment. We found that Zfp212 is non-epistatic to Fancd2 (Figure 5F). In summary, using epistatic analysis in mESCs, we verified that Traip exhibited non-epistatic relationship to both the Neil3 and FA pathways for TMP-ICL repair as reported in human cells (32). Similar to Traip, Zfp212 was non-epistatic to both the Neil3 pathway and FA pathway for repairing TMP-ICL lesions. Our data suggest that Zfp212 likely acts upstream of both ICL repair pathways in collaboration with Traip.

### Zfp212 and traip suppress chromosome instability in response to ICL-forming agents in collaboration with FA and/or neil3 pathways

**Figure 6.**
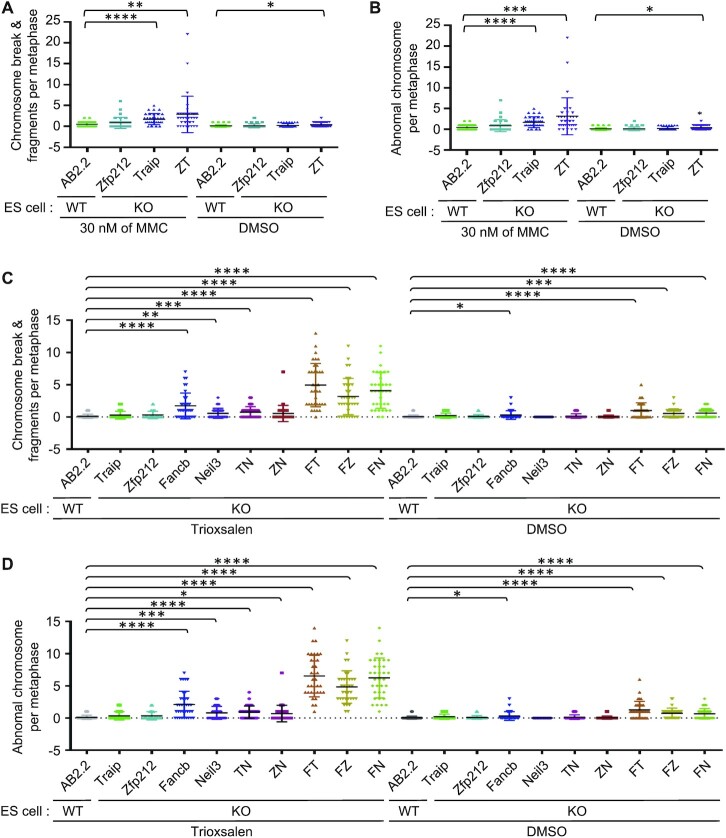
Zfp212 and Traip play important roles for maintaining chromosome integrity in response to ICL damages in collaboration with FA and Neil3 pathways. (A and B) MMC-induced chromosome instability in mESCs deleted for Zfp212, Traip or both. AB2.2 is a wild-type mESCs (control) and ZT-DKO is a mESCs deleted for both Zfp212 and Traip. (**A**) Chromosomal breaks and fragments with or without MMC in indicated mESCs. (**B**) Abnormal chromosomes with or without MMC in indicated mESCs. Abnormal chromosomes include chromosome breaks, fragments and radial chromosomes. (**C**, **D**) Chromosomal breaks & fragments and abnormal chromosomes with or without TMP in indicated mESCs, respectively. Cells were treated with TMP (0.03 ng/ml) following UVA, and then released for 20 hr. After that, cells were processed. Thirty-five metaphase cells were counted at the indicated condition. Data represent the mean ± SD. Unpaired *t* test was performed for statistics (Prism 8 software). *P< 0.05, **P< 0.01, ***P< 0.001, ^****^P< 0.0001. TN-DKO: Traip-Neil3 double knockout (DKO); ZN-DKO: Zfp212-Neil3 DKO; FT-DKO: Fancb-Traip DKO; FZ-DKO: Fancb-Zfp212 DKO; FN-DKO: Fancb-Neil3 DKO.

We showed that both HeLa cells depleted with ZNF212 and Zfp212 KO mESCs showed increased sensitivity to MMC. We observed only marginal increment of chromosome breaks and abnormal chromosomes in Zfp212 or Traip KO mESCs in response to MMC compared to WT mESCs (Figure 6A and B, Supplementary Figure S9A, and Supplementary Table S4). Unlike drug sensitivity and DNA damage response, chromosome instability was exacerbated when both *Zfp212* and *Traip* genes were deleted, implying that there might be undefined non-epistatic functions between Zfp212 and Traip in maintaining chromosome stability. MMC-induced formation of radial chromosomes was not significantly increased in Zfp212 KO or Traip KO mESCs although statistical meaningful difference was observed when both *Zfp212* and *Traip* genes were deleted (Supplementary Figure S10A).

To evaluate the relative contribution and genetic interactions of Zfp212, Traip, Fancb and Neil3 in suppressing chromosome instability, we performed epistatic analysis of chromosome spreads in gene knockout mESCs with or without TMP and MMC treatment. We found that Fancb deletion significantly increased the number of chromosome breaks, and that these were further increased by additional deletion of Zfp212, Traip or Neil3, respectively (Figure 6C and D, Supplementary Figure S9B and Supplementary Table S5). Additionally, TMP treatment significantly increased chromosomal aberrations in Fancb-Traip, Fancb-Zfp212, and Fancb-Neil3 double knockout mESCs as compared to single Fancb KO mESCs (Figure 6C and D, Supplementary Figures S9B and S10B and Supplementary Table S5), suggesting that these proteins participate in preventing spontaneous and TMP-induced chromosome instability, and that the FA pathway seems non-epistatic to Zfp212, Traip, or Neil3 for this function. The TMP-induced chromosome instability correlates with the cell survival (Figure 5), suggesting TMP-induced chromosome instability likely be the cause of cell death. Next, we measured MMC-induced chromosome instability in these knockout mESCs. Deletion of Traip, Zfp212 or Neil3 in Fancb KO mESCs significantly increased chromosome abnormalities following MMC treatment compared to Fancb KO mESCs, suggesting these proteins are important for suppressing MMC-induced chromosome aberrations (Supplementary Figure S11A–C and Supplementary Table S5).

### ZNF212 promotes the recruitment of NEIL3 to ICLs through a direct interaction

**Figure 7.**
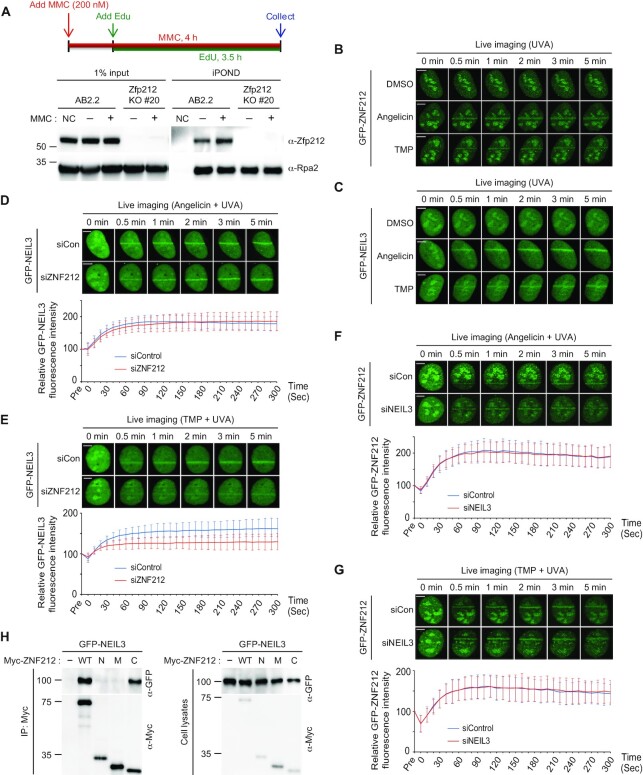
ZNF212 promotes NEIL3 recruitment to ICL lesions through interaction with NEIL3. (**A**) The localization of Zfp212 on nascent strand in mESCs by iPOND assay with and without MMC treatment. (**B**, **C**) U2OS cells were transfected with either GFP-ZNF212 (B) or GFP-NEIL3 (C). 24 h post-transfection, transfected cells were treated with DMSO, angelicin or TMP, and then treated with laser microirradiation. We examined the laser strip at each indicated time. (**D**–**G**) U2OS cells were treated with indicated siRNA. Next day, cells were transfected with GFP-NEIL3 (C and D) or GFP-ZNF212 (F and G) and treated with 2mM of thymidine. After 17 hr, cells were released in fresh medium for another 9 h. Following the second thymidine block (2 mM, 18 h), cells were released with fresh medium for 4 h and then treated with laser microirradiation in the presence of either angelicin or TMP. The initial intensity of region of interest (ROI) before bleaching was calculated as 100% in each cell, and then the average intensity of the laser stripes was plotted. For each experiment, ten cells were analyzed. Data represent the mean ± SD from two independent experiments. Scale bar, 5 μm. (H) 293T cells were co-transfected with plasmids encoding GFP-NEIL3 and Myc-ZNF212 WT or its deletion mutants (N, M and C). 24 h post-transfection, cell lysates with Benzonase were subjected to IP with anti-Myc bead, and then immunoblotted with the indicated antibodies.

It was reported that TRAIP is found in DNA replication forks and functions in replication dependent DNA repair pathways (42,43). Therefore, we asked that if ZNF212 also resides in DNA replication forks. To this end, we performed iPOND analysis in AB2.2 mESCs in the absence or presence of MMC, we found that Zfp212 was located at DNA replication forks in the absence or presence of damage (Figure 7A). Of note, TRAIP has been shown to promote recruitment of NEIL3 to psoralen-ICL lesions in human cells (32). Thus, to determine if the ZNF212 plays a role in NEIL3 recruitment to sites of DNA damage, we examined the accumulation of NEIL3 and ZNF212 at laser-induced lesion formed by TMP (ICLs) or angelicin (monoadducts, angelicin is an analog of psoralen that forms mono-adducts that are primarily repaired by nucleotide excision repair) (44). We found that ZNF212 and NEIL3 were recruited to DNA lesions induced by either TMP or angelicin (Figure 7B and C, Supplementary Figure S12A and B) in U2OS cells. To investigate a role of ZNF212 in the replication-coupled ICL repair pathway, we performed microirradiation experiments with U2OS cells in S phase: microirradiation experiments were conducted with PCNA or cyclin A as S phase markers. As shown in the Supplementary Figure S12C–F, GFP-ZNF212 or GFP-NEIL3 co-localized with γH2AX in BrdU-, angelicin- or TMP-induced microirradiation assay in S phase. Next, using microirradiation, we asked if ZNF212 affects NEIL3 recruitment to the laser stripes induced by angelicin or TMP. We found that depletion of ZNF212 impaired NEIL3 translocation to the sites of DNA damage in the presence of TMP, but not in the presence of angelicin (Figure 7D and E). This observation suggests that ZNF212 specifically promotes NEIL3 recruitment to ICL lesions. Interestingly, depletion of NEIL3 had no specific impact on the recruitment of ZNF212 with treatment of angelicin or TMP (Figure 7F and G), suggesting that ZNF212 might function upstream of NEIL3. Next, we measured kinetics of the recruitment of NEIL3 and ZNF212 to laser stripes upon microirradiation. Both GFP-NEIL3 and mCherry-ZNF212 were found to localize to and then dissociate from DNA-damage sites with similar kinetics (Supplementary Figure S12G). Since ZNF212 has a role in TMP-ICL repair and influences NEIL3 recruitment to ICL lesions, we asked if ZNF212 directly interacts with NEIL3. To this end, GFP-NEIL3 was co-expressed with individual Myc-ZNF212-WT, -N, -M and -C in 293T cells, followed by immunoprecipitation and immunoblotting analysis. We found that full length ZNF212 and the C-terminal domain interacted with NEIL3 (Figure 7H). To confirm direct interaction between ZNF212 and NEIL3, we purified recombinant Flag-ZNF212 and HA-NEIL3 (Supplementary Figure S12H and I) and performed immunoprecipitation with increasing amounts of HA-NEIL3. This experiment confirmed the association between ZNF212 or ZNF212-C and NEIL3 is a bona fide direct interaction (Supplementary Figure S12J and K). We then tested whether expression of siRNA resistant ZNF212-WT or ZNF212 mutants rescued impaired NEIL3 accumulation on laser stripes in cells treated with siRNA against ZNF212. As expected, we found that the expression of ZNF212-WT and the ZNF212-C mutant rescued the recruitment of NEIL3 to laser stripes in HeLa cells depleted with endogenous ZNF212, while ZNF212-M failed to so (Supplementary Figure S12L). The expression of siRNA resistant ZNF212-N also weakly rescued the translocation compared to that of ZNF212-WT or -C, (Supplementary Figure S12L), suggesting a possible existence of indirect association between ZNF212-N and NEIL3 through unknown factors. Consistent with previous report (32), we noticed that, in U2OS cells, depletion of MCM7 merely affects recruitment of NEIL3 on the laser stripes induced by TMP (Supplementary Figure S13) although TRAIP-mediated ubiquitination of CMG complex plays an important role in NEIL3 recruitment in Xenopus egg extract (17). Understanding molecular basis of the discrepancy remains elusive. In summary, these data suggest that, besides the role of ZNF212 for MMC-ICL repair, ZNF212 seems to repair TMP-induced ICLs via promoting NEIL3 recruitment to ICL lesions through direct interaction with NEIL3.

## DISCUSSION

TRAIP is involved in the DNA damage response (DDR) and multiple DNA repair pathways and physiological importance of TRAIP functions has been manifested by the identification of biallelic mutations of TRAIP gene in individuals presented with primordial dwarfism (29). TRAIP executes versatile tasks through the E3 ubiquitin ligase activity or interaction with factors implicated in genome maintenance (17,27–30). However, how TRAIP carries out those functions largely remains elusive. In this study, we identified ZNF212 as an interacting partner for TRAIP. Depletion of ZNF212 results in impaired recruitment of TRAIP to the sites of DNA damage and similarly siRNA-mediated TRAIP silencing leads to defects in ZNF212 localization to DNA lesions, suggesting that ZNF212 and TRAIP form a complex to move to sites of DNA damage. Experimental evidence proves that ZNF212 is involved in DNA damage response, homologous recombination and ICL repair pathways, in which TRAIP is implicated. Together with TRAIP, ZNF212 appears to function upstream of both the NEIL3 and FA pathways for the ICL repair. Interestingly, we found that ZNF212 directly interacts with NEIL3 and promotes NEIL3 recruitment on TMP-ICL lesions although further studies are required to understand how TRAIP, ZNF212 and CMG complex manage the NEIL3 localization to DNA lesions.

In order to understand genetic interaction among *Zfp212*, *Traip*, *Neil3* and *FA* genes in the ICL repair pathway, we utilized gene knockout mESC lines which allow us to analyze it with clearer genetic background. Similar to the defects identified in human cells depleted with TRAIP or ZNF212, Traip and Zfp212 knockout mESC lines recapitulate all the defects, including DDR, HR and ICL repair, which prompt us to generate additional gene knockout mESC lines. In consistent with the recent findings in human cells (32), we validated non-epistatic relationship of Traip to both Neil3 and FA pathways in mESCs to TMP-ICL repair, implying that TRAIP likely plays roles upstream of both pathways. In the current studies, epistatic analysis using Traip, Zfp212, Neil3, Fancb, Fancd2 single knockout and a series of double knockout mESCs clearly showed Zfp212 is non-epistatic to the Neil3 pathway. In addition, genetic interaction between Zfp212 and Fancb or Fancd2 showed enhanced sensitivity to TMP-ICL sensitivity, suggesting Zfp212 is also non-epistatic to FA pathway for TMP-ICL repair. Collectively, these findings suggest that ZNF212 and TRAIP likely function together upstream of both pathways for ICL repair. It was reported that the MCM7 subunit of the replicative helicase was identified as a substrate for TRAIP in *Xenopus* egg extract (17). When the CMG complex encounters ICLs, TRAIP ubiquitinates MCM7 and directs it to either the FA pathway or NEIL3-dependent ICL repair pathway. However, although iPOND analysis showed that Zfp212 is located at replication forks, we were not able to observe MCM7 ubiquitination either by Traip or Zfp212 in mESC lines (data not shown). We also observed that depletion of MCM7 did not significantly affect NEIL3 accumulation on the laser stripes in HeLa cells (Supplementary Figure S13), which is consistent with the previous report (32). The discrepancy might be due to the different model systems. *Xenopus* egg extract has been thought to be one of the best model system to study DNA replication as it contains all the proteins required for DNA replication. DNA added to the extract efficiently initiates replication to make DNA duplicates. However, human cells are hardly representing S-phase and, more importantly, the amount of ICL damage due to the treatment of MMC or TMP is relatively small compared to non-damaged DNA fraction, which might be the reason to observe unloading of CMG complex using iPOND assay. As up to 60% of exponentially growing mESCs are in S-phase (45,46), possibly the genetically modified mESC lines will be good model system to study replication associated DNA repair pathways although technically improvement will be required.

Here we also showed non-epistatic relation between NEIL3 and FA pathway for TMP-ICL repair as previously reported (17,32), but we observed that FA deficiency (Fancb and Fancd2 KO mESCs) led to much more sensitivity to TMP as compared to Neil3 deficiency in mESCs. In addition, we showed FA deficiency dramatically increases TMP-induced chromosome instability compared to Neil3 KO mESCs. These data suggest that FA pathway plays major roles for TMP-ICL repair for cell survival and genome maintenance in mESCs. Besides ICL repair, FA proteins also have additional roles in replication fork maintenance that may account for the additional increase for chromosome instability. The usage of different cell lines is likely causal for this discrepancy as suggested by other groups (17,32). FA pathway is active mainly in the S-phase and that the majority of ICLs are repaired in a replication-dependent manner (47). mESCs were shown to have prolonged S-phase in asynchronous population, leading to up to 40–60% of cells in S phase (45,46). Fancd2 was found to be highly expressed in mESCs compared to other somatic tissues (48). These intrinsic characteristics of mESCs likely renders mESCs more dependent on FA pathway for repairing ICLs damages than on Neil3 pathway for cell survival as compared to somatic cells. It is worth to note that, as in the previous reports (32), damage induced FANCD2 monoubiquitination was observed in Traip KO and Zfp212 KO mESCs. However, deletion of either Traip or Zfp212 in mESCs did not increase the protein expression level of Fancd2. These results are different from the previous reports in human HeLa cells showing that depletion of TRAIP increases the expression of FANCD2 in response to psoralen-ICLs. Again, the discrepancy might due to the difference of mammalian cells examined. Another possible explanation would be the different molecular effects of acute reduction and long-term adaptation. In order to establish gene KO mESC line, it takes long time and thus during selection period, the cells might change the cellular processes to survive against various genomic insults. On the other hand, siRNA-mediated gene silencing is an acute process, in which there is no enough time for cells to adapt themselves against depletion of specific genes. Of note, it was reported that the recruitment of FA core and FANCI-D2 complex to the sites of ICLs are replication independent, while that of BRCA-related FA proteins are replication dependent (49). Therefore, cells might be sensitive to MMC or TMP-ICLs as the cells cannot completely repair the ICLs in the absence of TRAIP, but monoubiquitination of FANCD2 is normal as FANCI-D2 complex senses ICLs in the replication independent manner.

The immunoprecipitation data indicates that N- and C-terminus of ZNF212 domains are associated with TRAIP (Figure 1F–H). However, as shown in the Figure 2E, the fluorescent intensity of ZNF212-N and ZNF212-C stripes were relatively lower compared to that of ZNF212 wild type although ZNF212-WT, ZNF212-N and ZNF212-C accumulated on the laser stripes in most of the cells. Further studies showed that the ZNF212-ΔM showed similar level of recruitment to that of ZNF212-WT, suggesting that both N- and C-terminus of ZNF212 are required for full recruitment of TRAIP to the sites of DNA damage (Figure 2E). Currently we do not understand roles of middle region of ZNF212 in TRAIP associated genome maintenance functions. However, interestingly, expression of either ZNF212-N or ZNF212-C in cells depleted with endogenous ZNF212 rescues MMC, CPT and HU sensitivity (Figure 4A–C), and also damage induced foci formation of RAD51 and RPA2 to the level of ZNF212-WT (Figure 4E–F). Together with lower recruitment of ZNF212-N and ZNF212-C to the sites of DNA damage, these findings suggest that having a partial activity of TRAIP might be enough to play a specific role in DNA damage response and DNA repair. Further studies will be needed to have clear answer how ZNF212 regulates the TRAIP function. Besides the E3 ligase function, TRAIP plays a diverse roles in genome maintenance through interaction with multiple binding partners. Specifically, TRAIP looks to interact with either ZNF212 or RNF20/40 through the same domain (D5 in the Figure 1E). As the RNF20/40 has been implicated in homologous recombination (30,50), sharing the same domain for different interaction raises the possibility that ZNF212 and RNF20/40 might modulate the versatile functions of TRAIP in HR and ICL repair. Further studies will be required to understand how multiple functions of TRAIP are regulated in different cellular pathways.

It was reported that short ubiquitin chains of CMG complex promote NEIL3 localization at ICL lesions in Xenopus egg extract (17), and later in human cells NEIL3 recruitment to psoralen-ICL is PARP-dependent (32). Here, we showed that ZNF212 is associated with NEIL3 and depletion of ZNF212 significantly reduced NEIL3 accumulation on TMP-induced laser stripes (Figure 7). From the immunoprecipitation analysis, ZNF212-C domain is responsible for ZNF212 and NEIL3 interaction. However, interestingly, we observed that expression of ZNF212-N in cells depleted with endogenous ZNF212 rescued NEIL3 accumulation on TMP-induced laser stripes. One possible explanation would be that ZNF212-N promote TRAIP-dependent ubiquitination at replication forks which can rescue NEIL3 recruitment independent of an interaction with ZNF212. Further studies will be required to prove the hypothesis and also to understand molecular basis of TRAIP recruitment to the ICL damage.

Mutations in TRAIP were found in patients suffering from primodial dwarfism (29) and the recombinant protein carrying the patient mutation (R18C) still forms short ubiquitin chains on MCM7 but fails to repair cisplatin-ICL repair while promoting TMP-ICL repair in *Xenopus* system (17). Consistent with previous studies, the R18C mutation could not complement MMC sensitivity in our study (Supplementary Figure S6K). Surprisingly, the *Traip* knockout in mice and Drosophila causes embryonic lethality suggesting that Traip has an essential role for development (24,25). Since the R18C mutation of *TRAIP* that is defective in ICL repair does not cause embryonic lethality in human, it is possible that TRAIP may have other essential function during embryonic development in DNA replication and mitosis. TRAIP localizes to DNA replication forks in the absence of exogenous DNA damage. The PIP box in TRAIP suggests that TRAIP is localized to DNA replication forks through PCNA interaction. Interestingly, TRAIP lacking the PIP box complements MMC sensitivity in human cells (27) and mESCs (Supplementary Figure S6L). Thus, TRAIP may have an essential role for DNA replication during embryonic development. Alternatively, the residual activity of the R18C mutation allows slow kinetic repair of ICL, which might be enough to repair DNA damage during embryonic development and only causes primordial dwarfism phenotype after birth.

In summary, we demonstrates that, as a novel interaction partner of TRAIP, ZNF212 plays important roles in DNA damage signaling and HR for cell survival and genome maintenance, and likely act upstream of both NEIL3 and FA pathways for ICL repair. In addition, TRAIP appears to function as important factor for ICL repair as a regulatory factor upstream of both the NEIL3 and FA pathways in mESC lines. As a master regulator, TRAIP functions in ICL repair has just identified. Our findings together with mESC lines used in this study will be informative to understand molecular basis of the ICL repair pathways in detail.

## DATA AVAILABILITY

All the materials, methods and raw data generated in this study are available upon request. All the data supporting the findings are available in online version. pX330 plasmid is available in Addgene (#42230). Human ZNF212 plasmid was purchased Korea Human Gene Bank (hMU003418).

## Supplementary Material

gkac1226_Supplemental_File
