# Supplementary material for: ZNF212 promotes genomic integrity through direct interaction with TRAIP
Source: Nucleic Acids Res. 2023 Jan 3;51(2):631–49. doi: 10.1093/nar/gkac1226 (PMC9881131; doi:10.1093/nar/gkac1226)
Supplement: gkac1226_Supplemental_File [file gkac1226_supplemental_file.pdf]

SUPPLEMENTARY DATA for

ZNF212 promotes genomic integrity through direct interaction with TRAIP

Hee Jin Chung[1,4], Joo Rak Lee[1,4], Tae Moon Kim[1,2,4], Soomi Kim[1,4], Kibeom Park[1,4], Myung-Jin Kim[3], Eunyoung Jung[3], Subin Kim[1], Eun A Lee[2], Jae Sun Ra[2], Sunyoung Hwang[2], Ja Yil Lee[1], Orlando D. Schärer[1,2], Yonghwan Kim[3,*], Kyungjae Myung[1,2,*], and Hongtae Kim[1,2,*]

[1]Department of Biological Sciences, Ulsan National Institute of Science and Technology, Ulsan 44919, Republic of Korea. [2]Center for Genomic Integrity Institute for Basic Science (IBS), Ulsan 44919, Republic of Korea. [3]Department of Biological Sciences and Research Institute of Women's Health, Sookmyung Women's University, Seoul 04310, Republic of Korea.

[4]These authors contributed equally to this work

Supplementary Figure S1 to S13

Supplementary Table S1 to S5

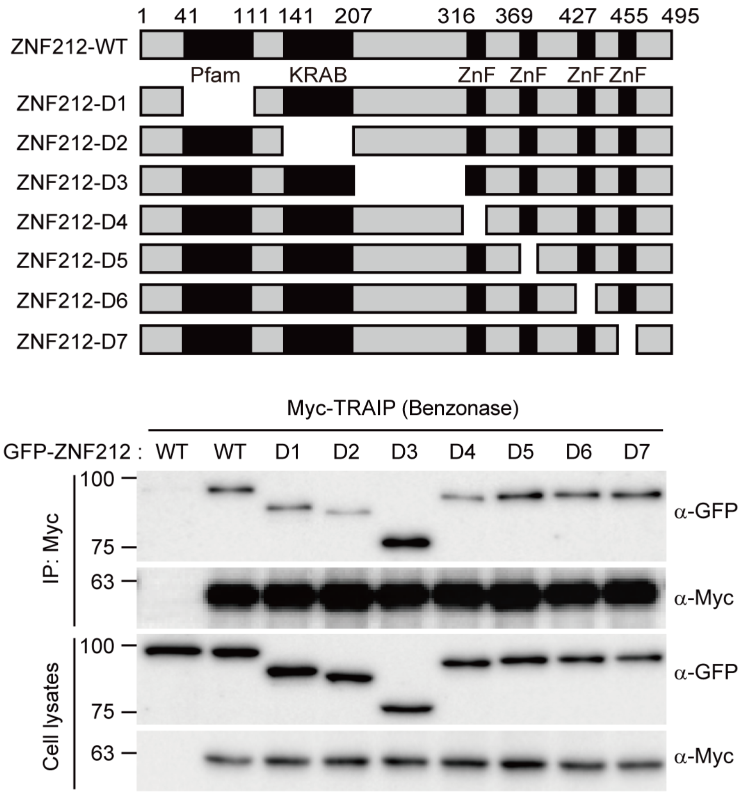

Supplementary Figure S1. Myc-TRAIP interacted with all of deletion mutants for GFP-ZNF212. The diagram of ZNF212 WT and deletion mutants (ZNF212 D1 to D7) (upper panel). The indicated plasmids were co-transfected into 293T cells. After 48 hr, transfected cell lysates with benzonase were immunoprecipitated using anti-Myc bead, and then subjected to Western blotting analysis using the indicated antibodies (bottom panel).

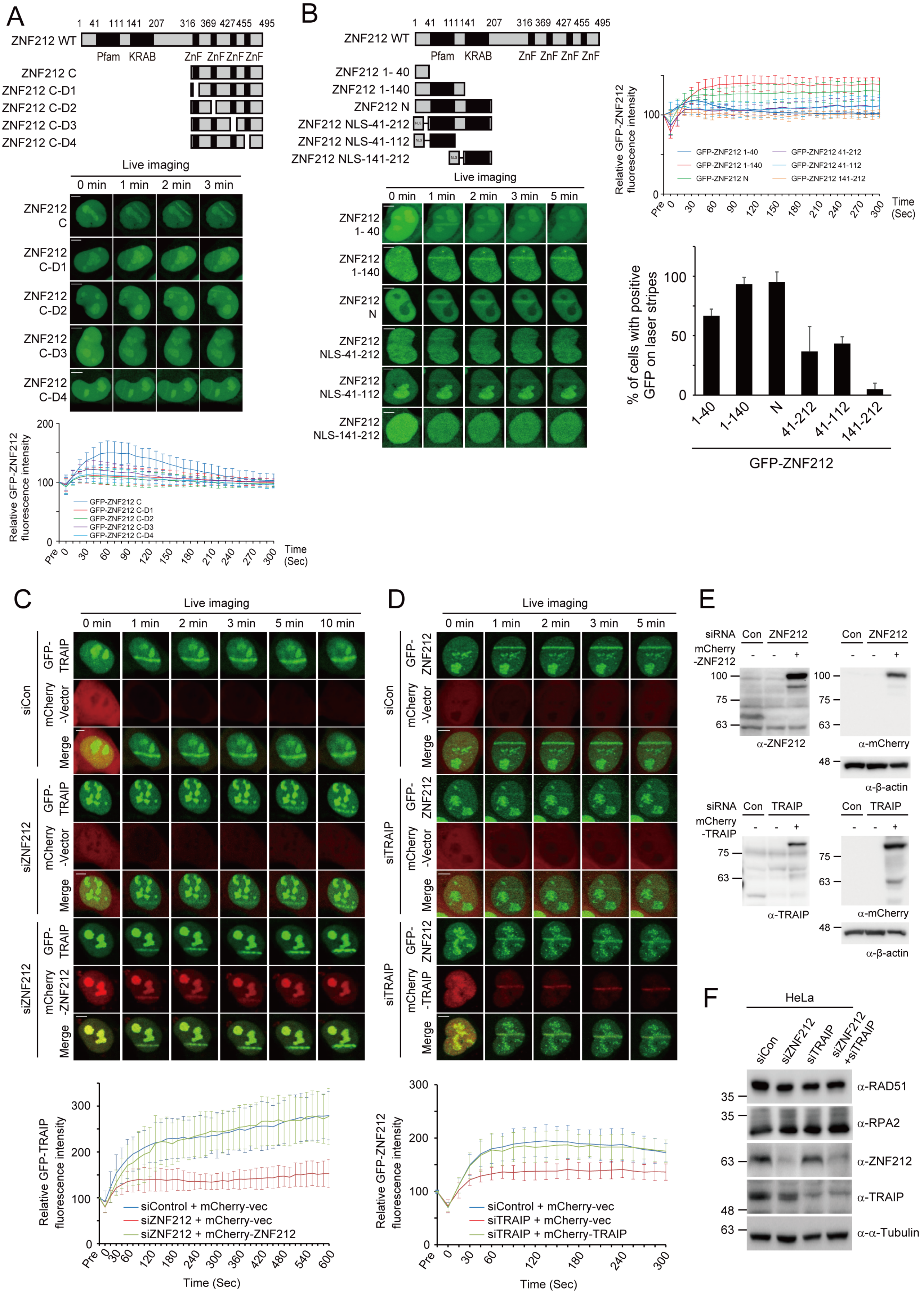

A
ZNF212 WT
1 41 111 141 207 316 369 427 455 495
Pfam KRAB ZnF ZnF ZnF ZnF
ZNF212 C
ZNF212 C-D1
ZNF212 C-D2
ZNF212 C-D3
ZNF212 C-D4
Live imaging
0 min 1 min 2 min 3 min
Relative GFP-ZNF212 fluorescence intensity
GFP-ZNF212 C
GFP-ZNF212 C-D1
GFP-ZNF212 C-D2
GFP-ZNF212 C-D3
GFP-ZNF212 C-D4
Time (Sec)
B
ZNF212 1- 40
ZNF212 1-140
ZNF212 N
ZNF212 NLS-41-212
ZNF212 NLS-41-112
ZNF212 NLS-141-212
Live imaging
0 min 1 min 2 min 3 min 5 min
GFP-ZNF212 1-40
GFP-ZNF212 1-140
GFP-ZNF212 N
GFP-ZNF212 41-212
GFP-ZNF212 41-112
GFP-ZNF212 141-212
% of cells with positive GFP on laser stripes
1-40 1-140 N 41-212 41-112 141-212
GFP-ZNF212
C
Live imaging
0 min 1 min 2 min 3 min 5 min 10 min
siCon
siZNF212
GFP-TRAIP
mCherry -Vector
mCherry -ZNF212
Merge
Relative GFP-TRAIP fluorescence intensity
siControl + mCherry-vec
siZNF212 + mCherry-vec
siZNF212 + mCherry-ZNF212
Time (Sec)
D
Live imaging
0 min 1 min 2 min 3 min 5 min
siCon
siTRAIP
GFP-ZNF212
mCherry -Vector
mCherry -TRAIP
Merge
Relative GFP-ZNF212 fluorescence intensity
siControl + mCherry-vec
siTRAIP + mCherry-vec
siTRAIP + mCherry-TRAIP
Time (Sec)
E
siRNA Con ZNF212
mCherry -ZNF212
α-ZNF212
α-mCherry
α-β-actin
siRNA Con TRAIP
mCherry -TRAIP
α-TRAIP
α-mCherry
α-β-actin
F
HeLa
siCon siZNF212 siTRAIP siZNF212 +siTRAIP
α-RAD51
α-RPA2
α-ZNF212
α-TRAIP
α-α-Tubulin

Supplementary Figure S2. TRAIP and ZNF212 interaction promotes recruitment of both proteins to the sites of DNA damage. (A) ZNF212 translocation to sites of DNA damage is dependent on all of each ZnF at C-terminus. HeLa cells were transiently transfected with GFP-ZNF212C or each ZnF-deletion mutant plasmids (ZNF212 C-D1 to C-D4). After 24 hours, microirradiation assay was performed in the presence of BrdU. Relative GFP intensity was determined at the indicated time points of each GFP-ZNF212 mutants (bottom panel) (B) HeLa cells were transfected with GFP-ZNF212N or individual mutants (ZNF212 1-40, 1-140, ZNF212 N, ZNF212 NLS-41-212, ZNF212 NLS-41-112, ZNF212 NLS-141-212). After 24 hours, laser microirradiation was performed in the presence of BrdU. Relative GFP intensity was determined at the indicated time points of each GFP-ZNF212 mutants (right upper panel). The percentages of cells with GFP positive on the laser stripes were presented in bar graph. For each experiment, ten cells for each group were analyzed. Data represent the mean ± SEM from two independent experiments. (C) HeLa cells treated with either control or ZNF212 siRNAs. After 48 hr, GFP-TRAIP was transiently transfected together with either mCherry-empty vector or mCherry-ZNF212. After 24 hours, laser microirradiation assay was performed in the presence of BrdU. (D) HeLa cells treated with either control or TRAIP siRNAs. After 48 hr, GFP-ZNF212 was transfected with either mCherry-empty vector or mCherry-TRAIP. After 24 hours, laser microirradiation assay was performed in the presence of BrdU. The initial intensity of region of interest (ROI) before bleaching was calculated as 100% in each cell, and then the average intensity of the laser stripes was plotted. For each experiment, ten cells were analyzed. Data represent the mean ±SD from two independent experiments. Scale bar, 5 µm. (E) Western blot analysis was performed to compare the protein expression level between endogenous ZNF212 or TRAIP and transiently expressed mCherry-ZNF212 or mCherry-TRAIP for the supplementary figure S2C and S2D. (F) Western blot analysis for RAD51, RPA2, ZNF212 or TRAIP protein level in HeLa cells treated with indicated siRNAs. Western blot analysis was performed using the indicated antibodies.

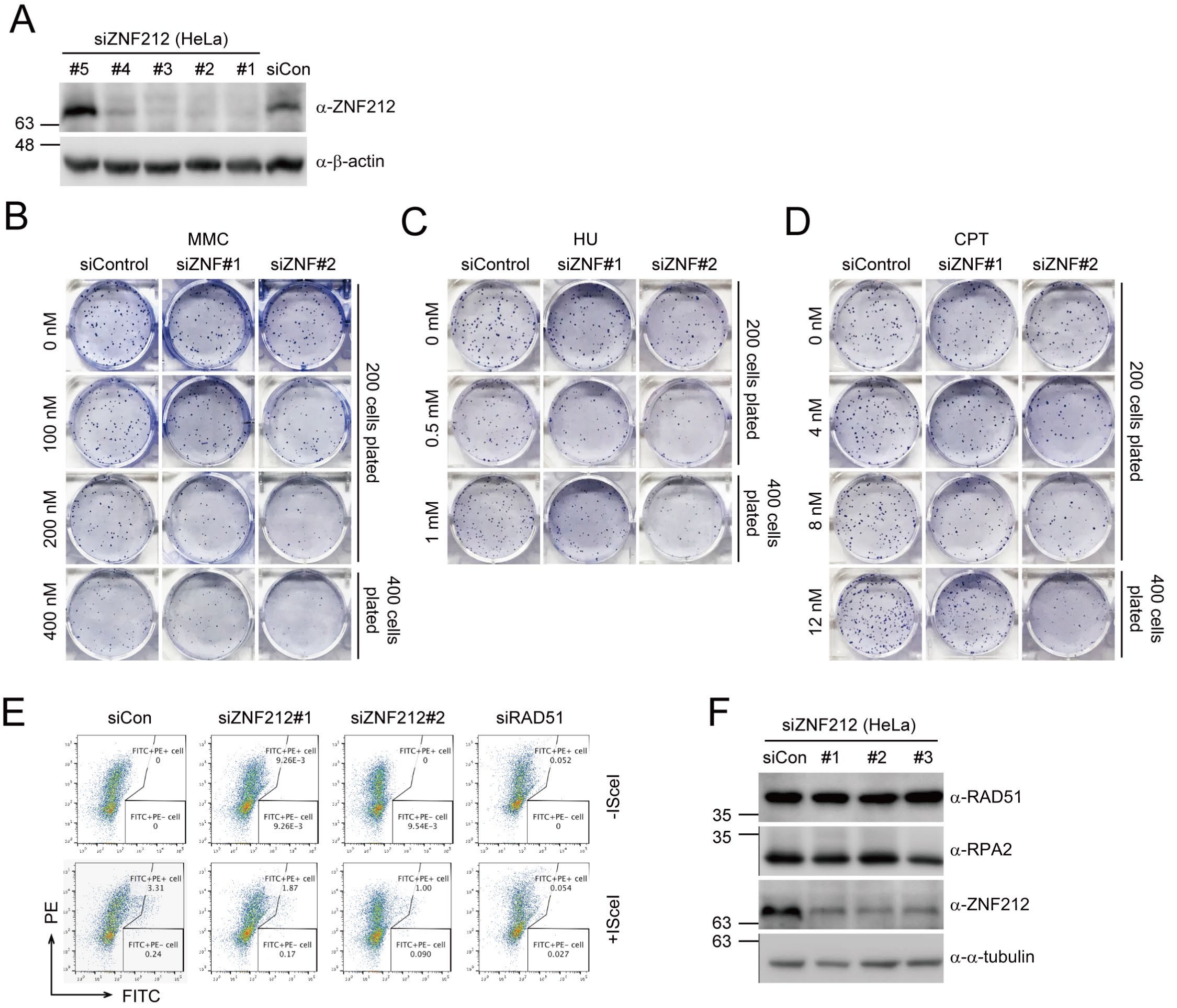

Supplementary Figure S3. Depletion of ZNF212 impairs DDR and HR. (A) Western blot analysis for ZNF212 in HeLa cells transfected with indicated siRNAs. (B - D) The representative images for clonogenic assay in response to DNA replication blockers, MMC, HU and CPT. HeLa cells were depleted with control or ZNF212 siRNAs, and then 200 or 400 cells were plated and treated with indicated doses of DNA damaging agents. The number of surviving colonies was counted 7 days after treatment of DNA damaging agents. (E) Representative FACS plots for DR-GFP based HR assays in ZNF212-depleted U2OS cells. (F) Western blot analysis for RAD51 or RPA2 protein level in HeLa cells transfected with indicated siRNAs. Western blot analysis was performed using the indicated antibodies.

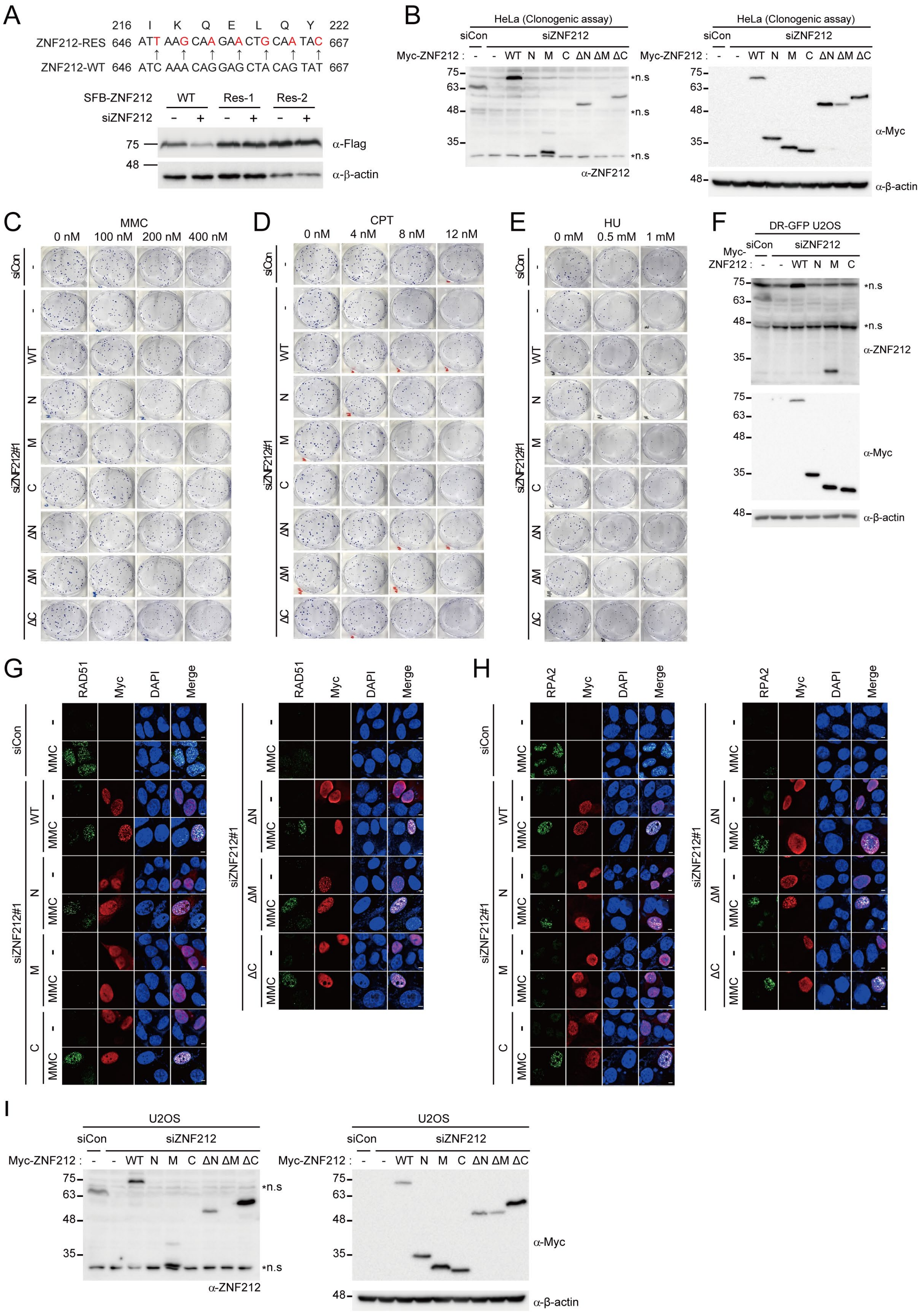

A
216 I K Q E L Q Y 222
ZNF212-RES 646 ATT AAG CAA GAA CTG CAA TAC 667
ZNF212-WT 646 ATC AAA CAG GAG CTA CAG TAT 667
SFB-ZNF212
WT
Res-1
Res-2
siZNF212
α-Flag
α-β-actin
B
HeLa (Clonogenic assay)
siCon
siZNF212
Myc-ZNF212 :
WT N M C ΔN ΔM ΔC
*n.s
α-ZNF212
α-Myc
α-β-actin
C
MMC
0 nM 100 nM 200 nM 400 nM
D
CPT
0 nM 4 nM 8 nM 12 nM
E
HU
0 mM 0.5 mM 1 mM
siZNF212#1
F
DR-GFP U2OS
G
RAD51
Myc
DAPI
Merge
MMC
H
RPA2
I
U2OS

Supplementary Figure S4. ZNF212 is implicated in DNA repair and DNA damage response. (A) The sequences of the siRNA-resistant ZNF212 WT cDNA showing the silent mutation (ZNF212-RES). For protein expression of ZNF212-RES, HEK293T cells were transfected siRNA for 24 hr and were transfected ZNF212 plasmid as indicated for 24 hr. And then whole cell lysates were prepared for western blotting to confirm that the ZNF212-RES is resistant to the ZNF212 siRNA treatment. (B) Comparison of expression level of endogenous and exogenous wildtype ZNF212, ZNF212-N, ZNF212-M, ZNF212-C, ZNF212-ΔN, ZNF212-ΔM, and ZNF212-ΔC in HeLa cell. Endogenous ZNF212 was depleted with siRNA against ZNF212 and siRNA resistant ZNF212 expression vectors were transfected transiently. Cells were harvested and the cell lysates were subject to Western blot analysis with indicated antibodies. ZNF212 antibody used in the Western blot was raised using amino-acids 230-309, which is only able to detect central region of the ZNF212-C (amino-acids 203-320). Therefore, ZNF212-N, ZNF212-C and ZNF212-ΔC were not detected (left panel). Relative expression level of ZNF212-N, ZNF212-C and ZNF212-ΔC was determined with anti-Myc antibody (right panel). (C - E) The representative images for rescuing the sensitivity of ZNF212-depleted HeLa cells to DNA replication blockers, MMC, CPT and HU. (F) Comparison of expression level for endogenous and exogenous wildtype ZNF212, ZNF212-N, ZNF212-M, and ZNF212-C in DR-GFP U2OS cell for HR assay. (G and H) The representative images for MMC-induced RAD51 and RPA2 foci formation. U2OS cells treated with either control or ZNF212 siRNAs and transfected with indicated plasmid. Cells were exposed to 0 or 500 nM of MMC for 16 hr, and then fixed and subjected to staining with indicated antibodies. Scale bar, 5 μm. (I) Comparison of expression level for endogenous and exogenous wildtype ZNF212, ZNF212-N, ZNF212-M, ZNF212-C, ZNF212- ΔN, ZNF212-ΔM, and ZNF212-ΔC in U2OS cell.

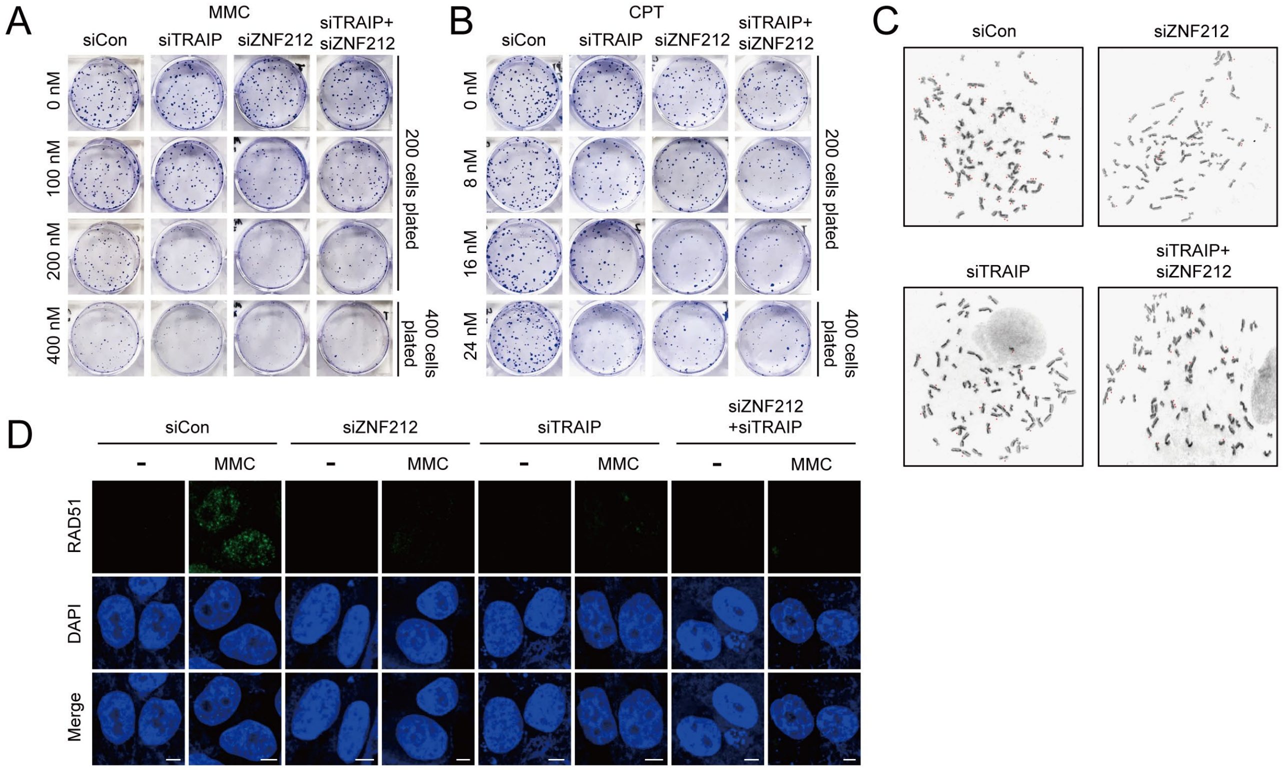

Supplementary Figure S5. ZNF212 is epistatic to TRAIP for cell survival in response to DNA replication blocking damages and HR. (A and B) The representative images for epistatic clonogenic assay in response to MMC and CPT. HeLa cells transfected with the indicated siRNA were plated and treated with indicated doses of each DNA damaging agent. The number of surviving colonies was counted 7 days after treatment of each DNA damaging agent. (C) The representative images for SCE in HeLa cells transfected with indicated siRNAs. Thirty-five metaphase cells were counted at the indicated condition. (D) The representative images for MMC-induced RAD51 foci formation. U2OS cells depleted with either control or ZNF212 siRNAs were exposed to 0 or 500 nM of MMC for 16 hr, and then fixed and subjected to staining with indicated antibodies. Scale bar, 5 μm.

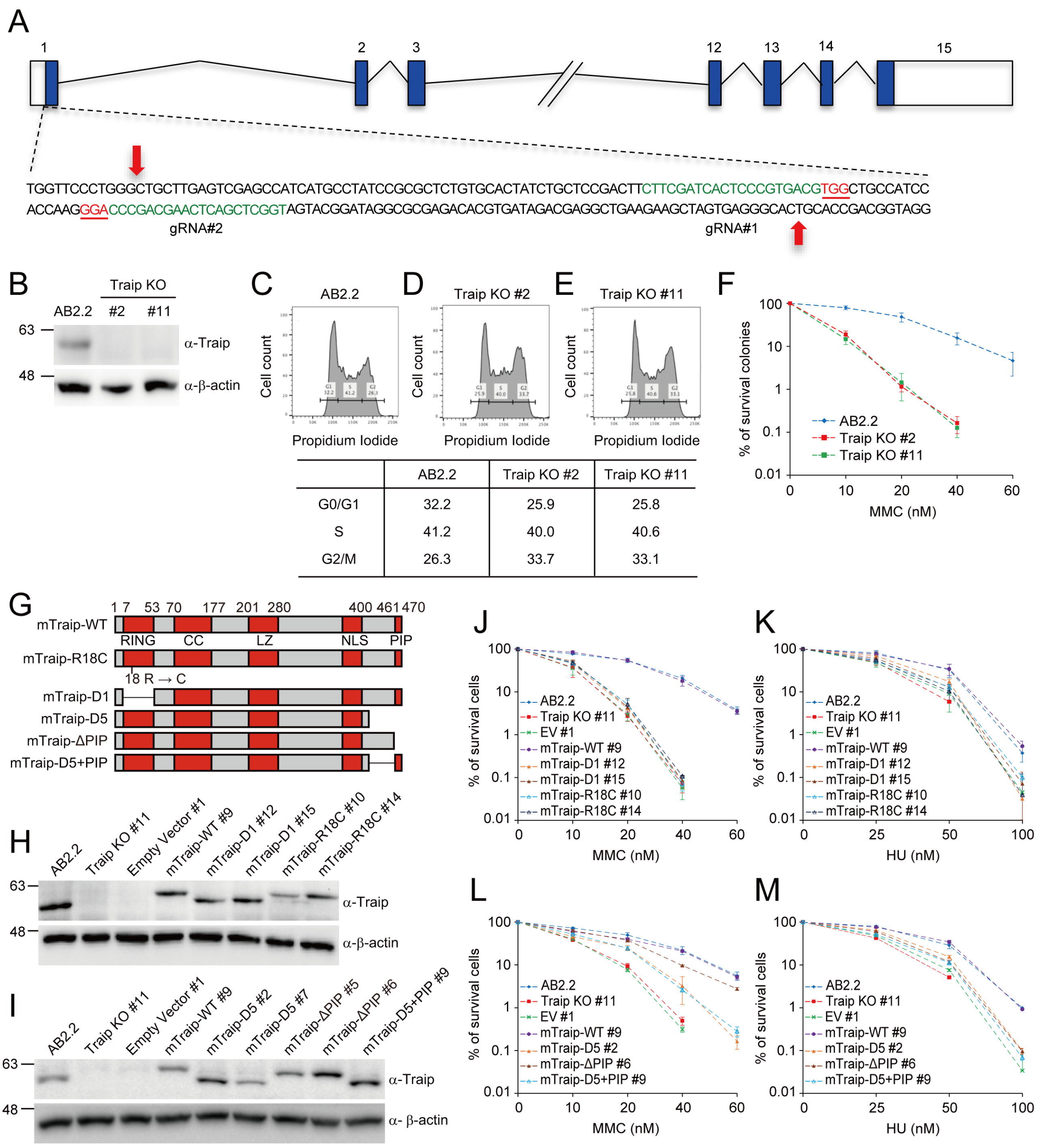

	AB2.2	Traip KO #2	Traip KO #11
G0/G1	32.2	25.9	25.8
S	41.2	40.0	40.6
G2/M	26.3	33.7	33.1

Supplementary Figure S6. The E3 ligase domain and Traip-protein interaction are required for repairing replication-blocking DNA damages or homologous recombination repair. (A) Mouse *Traip* genomic structure (chromosome 9) and guide RNAs in exon 1. (B) Western blot analysis for Traip protein in mESCs (AB2.2 and Traip KO) using indicated antibodies. AB2.2 is a wild type mESCs. (C - E) Cell cycle profile of AB2.2 and Traip KO mESCs. (F) Dose response curve to MMC in Traip KO mESCs. The graph represents the mean ± SEM from three independent experiments. (G) Schematic view of Traip domains and mutation tested in the study. (H and I) Western blot analysis for Traip protein in mESCs stably expressing the indicated *Traip* cDNAs (D1, R18C; D5, ΔPIP and D5 + PIP), respectively. (J - M) Dose response curve to MMC or HU in indicated mESCs. The graph represents the mean ± SEM from three independent experiments.

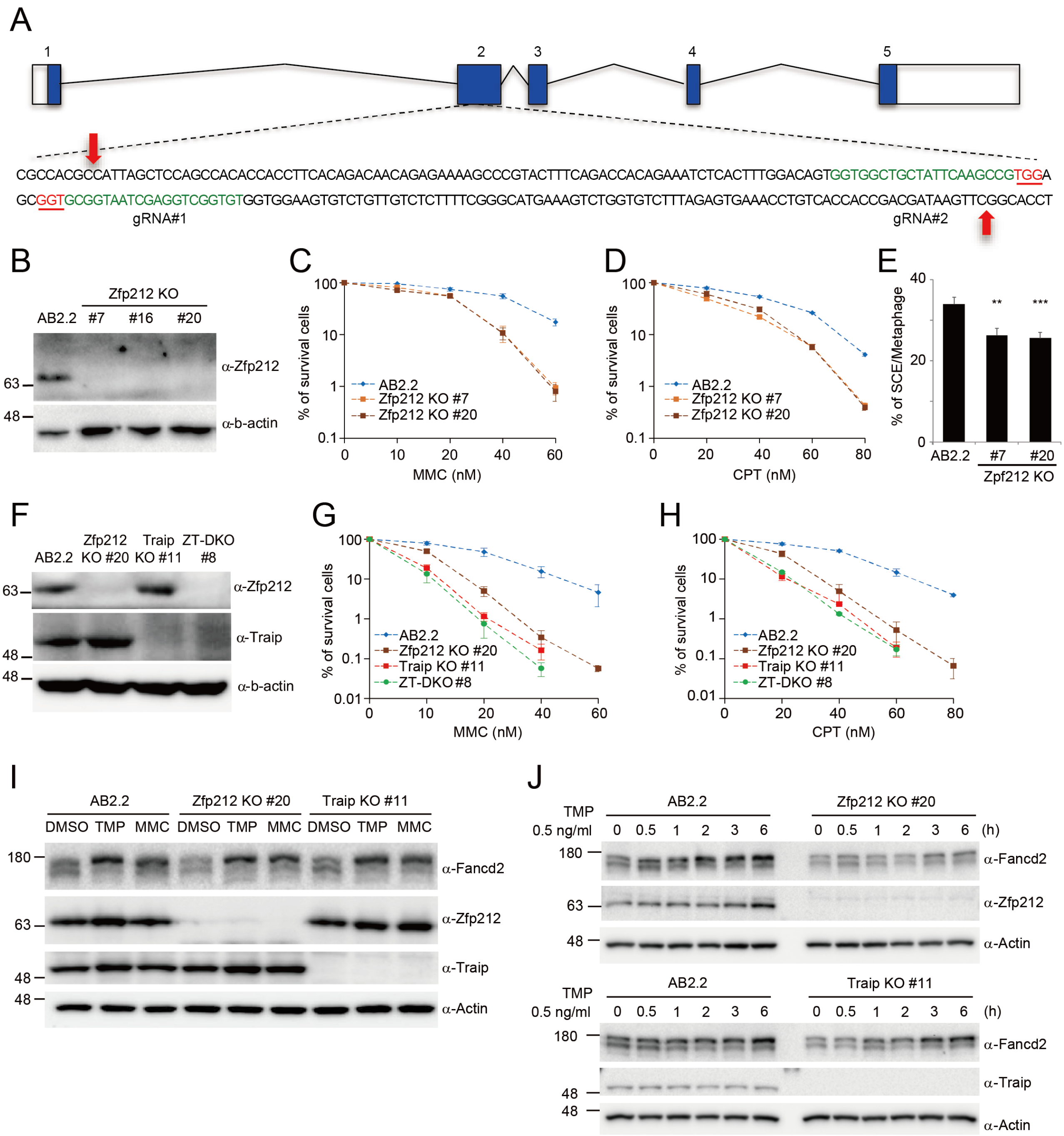
A
1
2
3
4
5
CGCCACGCCATTAGCTCCAGCCACACCACCTTCACAGACAACAGAGAAAAGCCCGTACTTTCAGACCACAGAAATCTCACTTTGGACAGTGGTGGCTGCTATTCAAGCCGTGGA
GCGGTGCGGTAATCGAGGTCGGTGTGGTGGAAGTGTCTGTTGTCTCTTTTCGGGCATGAAAGTCTGGTGTCTTTAGAGTGAAACCTGTCACCACCGACGATAAGTTCGGCACCT
gRNA#1
gRNA#2
B
Zfp212 KO
AB2.2 #7 #16 #20
63
48
α-Zfp212
α-b-actin
C
% of survival cells
MMC (nM)
AB2.2
Zfp212 KO #7
Zfp212 KO #20
D
% of survival cells
CPT (nM)
AB2.2
Zfp212 KO #7
Zfp212 KO #20
E
% of SCE/Metaphage
AB2.2
#7
#20
Zpf212 KO
**
***
F
AB2.2
Zfp212 KO #20
Traip KO #11
ZT-DKO #8
α-Zfp212
α-Traip
α-b-actin
G
% of survival cells
MMC (nM)
AB2.2
Zfp212 KO #20
Traip KO #11
ZT-DKO #8
H
% of survival cells
CPT (nM)
AB2.2
Zfp212 KO #20
Traip KO #11
ZT-DKO #8
I
AB2.2
Zfp212 KO #20
Traip KO #11
DMSO TMP MMC DMSO TMP MMC DMSO TMP MMC
180
63
48
48
α-Fancd2
α-Zfp212
α-Traip
α-Actin
J
TMP
0.5 ng/ml
AB2.2
Zfp212 KO #20
0 0.5 1 2 3 6 0 0.5 1 2 3 6 (h)
α-Fancd2
α-Zfp212
α-Actin
AB2.2
Traip KO #11
0 0.5 1 2 3 6 0 0.5 1 2 3 6 (h)
α-Fancd2
α-Traip
α-Actin

Supplementary Figure S7. Zfp212 is essential for cell survival in collaboration with Traip in mESCs. (A) Mouse *Zfp212* genomic structure (chromosome 6) and guide RNAs in exon 2. (B) Western blot analysis for Zfp212 protein level in mESCs (AB2.2 and Zfp212 KO) using indicated antibodies. (C and D) Dose response curve of indicated mESCs to MMC and CPT. The graph represents the mean ± SEM from three independent experiments. (E) Decreased SCE in Zfp212 KO mES cells as compared to AB2.2. Thirty-five metaphase cells were counted for the indicated condition. Data represent the mean ± SD. Unpaired t test was performed for statistics (Prism 8 software). ** $p < 0.01$, *** $p < 0.001$. (F) Western blot analysis for Zfp212 or Traip protein level in indicated mESCs. (G and H) Dose response curve of indicated mESCs to MMC and CPT. AB2.2, Zfp212 KO, Traip KO, and ZT-DKO (Zfp212 and Traip double KO) mES cells tested. The graph represents the mean ± SEM from three independent experiments. (I) Western blot analysis for Fancd2 protein and its monoubiquitylation level in response to 1 ng/ml TMP + UVA and 200 nM MMC in indicated mESCs using the indicated antibodies. (J) TMP induced time course of FANCD2 ubiquitination in Zfp212 or TRAIP KO mESCs. Cells were treated with 0.5 ng/ml TMP for 1 hr, then exposed to 6 kJ m^{-2} of 365 nm UVA light to photoactivate trioxsalen. The cells were washed twice with culture media, incubated at 37 °C for 10 min to remove unbound trioxsalen, then washed again and treated with 12 kJ m^{-2} UVA to convert trioxsalen monoadducts into ICLs. Then, cells were harvested each time point. Whole cell lysates immunoblotted with indicated antibodies.

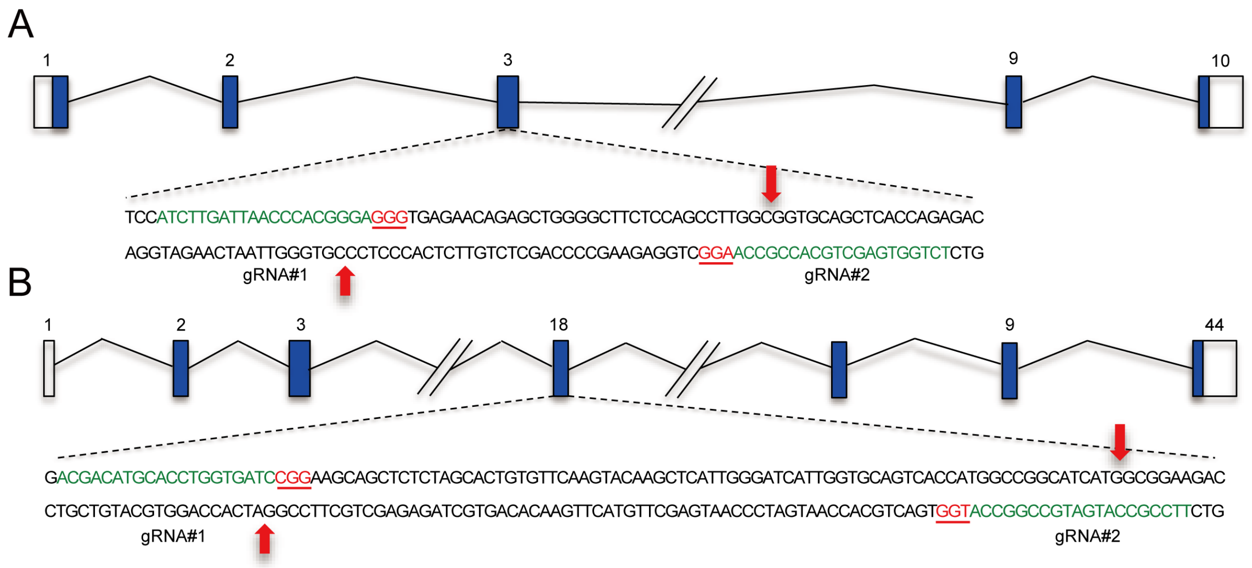

Supplementary Figure S8. The structure of genomic locus for *Neil3* and *Fancd2* and their gRNA information. (A) Mouse *Neil3* genomic structure (chromosome 8) and guide RNAs in exon 3. (B) Mouse *Fancd2* genomic structure and guide RNAs in exon 18.

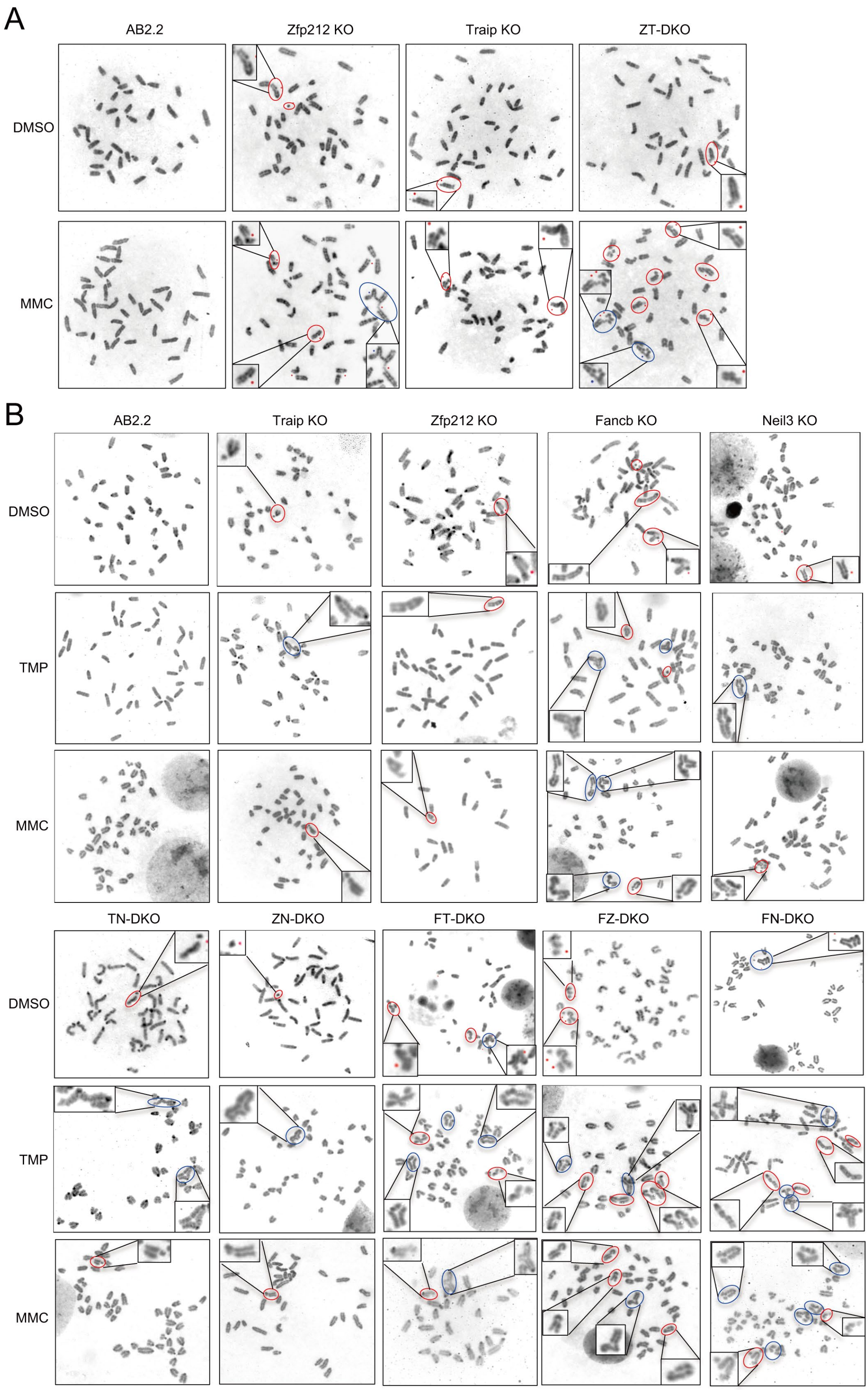
A
AB2.2
Zfp212 KO
Traip KO
ZT-DKO
DMSO
MMC
B
AB2.2
Traip KO
Zfp212 KO
Fancb KO
Neil3 KO
DMSO
TMP
MMC
TN-DKO
ZN-DKO
FT-DKO
FZ-DKO
FN-DKO
DMSO
TMP
MMC

Supplementary Figure S9. Zfp212 and Traip play important roles for maintaining genomic integrity in response to ICL damages in collaboration with FA and Neil3 pathways. (A and B) Representative images for spontaneous and ICL-induced chromosome instability in indicated mESCs.

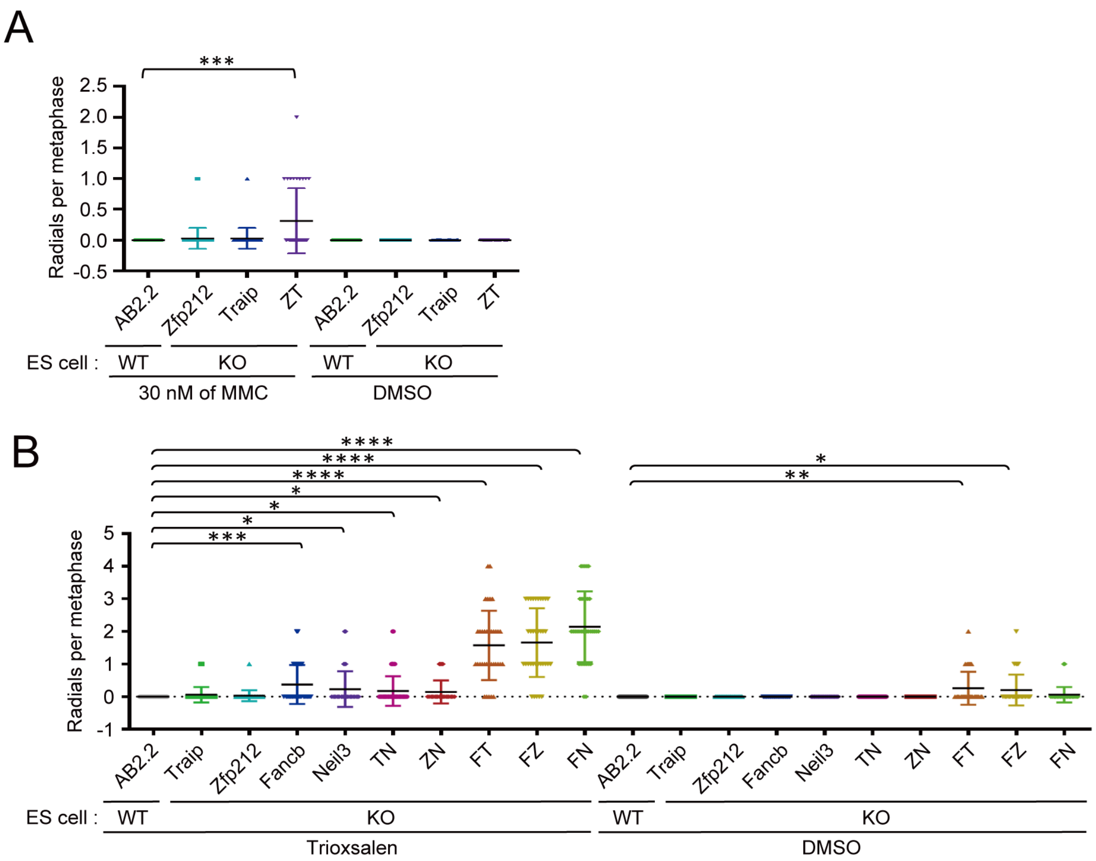

Supplementary Figure S10. Radial chromosomes induced by ICL damage in mESC lines. (A) Radial chromosomes with or without MMC in mESCs deleted for Zfp212, Traip or both. (B) Radial chromosomes with or without TMP in indicated mESCs. Thirty-five metaphase cells were counted at the indicated condition. Data represent the mean ± SD. Unpaired t test was performed for statistics (Prism 8 software). * $p < 0.05$, ** $p < 0.01$, *** $p < 0.001$, **** $p < 0.0001$. ZT: Zfp212-Traip double knockout (DKO); TN: Traip-Neil3 DKO; ZN: Zfp212-Neil3 DKO; FT: Fancb-Traip DKO; FZ: Fancb-Zfp212 DKO; FN: Fancb-Neil3 DKO.

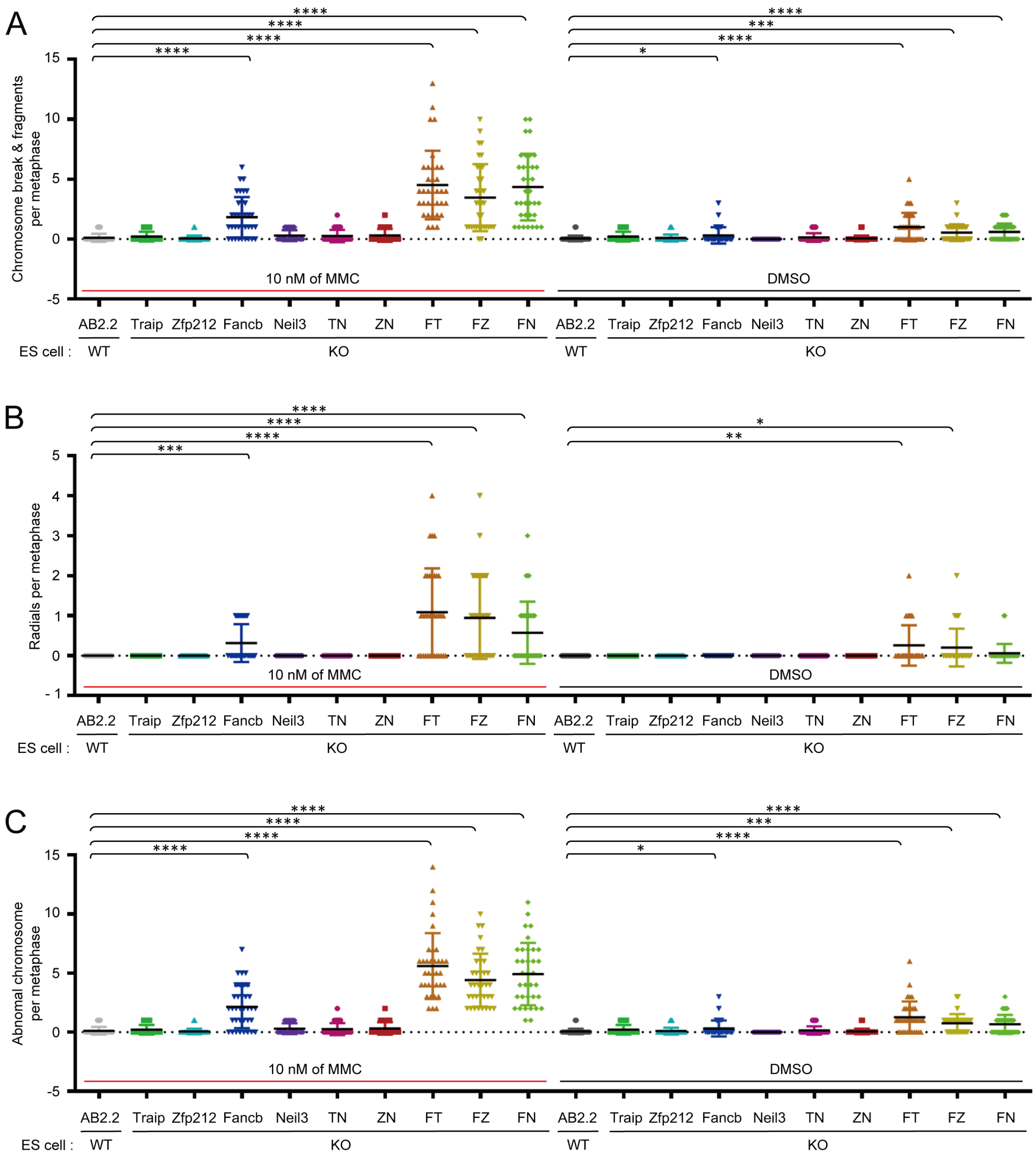
A
Chromosome break & fragments per metaphase
10 nM of MMC
DMSO
AB2.2 Traip Zfp212 Fancb Neil3 TN ZN FT FZ FN AB2.2 Traip Zfp212 Fancb Neil3 TN ZN FT FZ FN
ES cell : WT KO WT KO
B
Radials per metaphase
10 nM of MMC
DMSO
AB2.2 Traip Zfp212 Fancb Neil3 TN ZN FT FZ FN AB2.2 Traip Zfp212 Fancb Neil3 TN ZN FT FZ FN
ES cell : WT KO WT KO
C
Abnormal chromosome per metaphase
10 nM of MMC
DMSO
AB2.2 Traip Zfp212 Fancb Neil3 TN ZN FT FZ FN AB2.2 Traip Zfp212 Fancb Neil3 TN ZN FT FZ FN
ES cell : WT KO WT KO

Supplementary Figure S11. Chromosome instability induced by MMC in mESC lines. (A - C) Chromosomal breaks & fragments, radial chromosomes, and abnormal chromosomes with or without MMC in indicated mESCs, respectively. Abnormal chromosomes include chromosome breaks, fragments, and radial chromosomes. Thirty-five metaphase cells were counted at the indicated condition. Data represent the mean ± SD. Unpaired t test was performed for statistics (Prism 8 software). * $p < 0.05$, ** $p < 0.01$, *** $p < 0.001$, **** $p < 0.0001$.

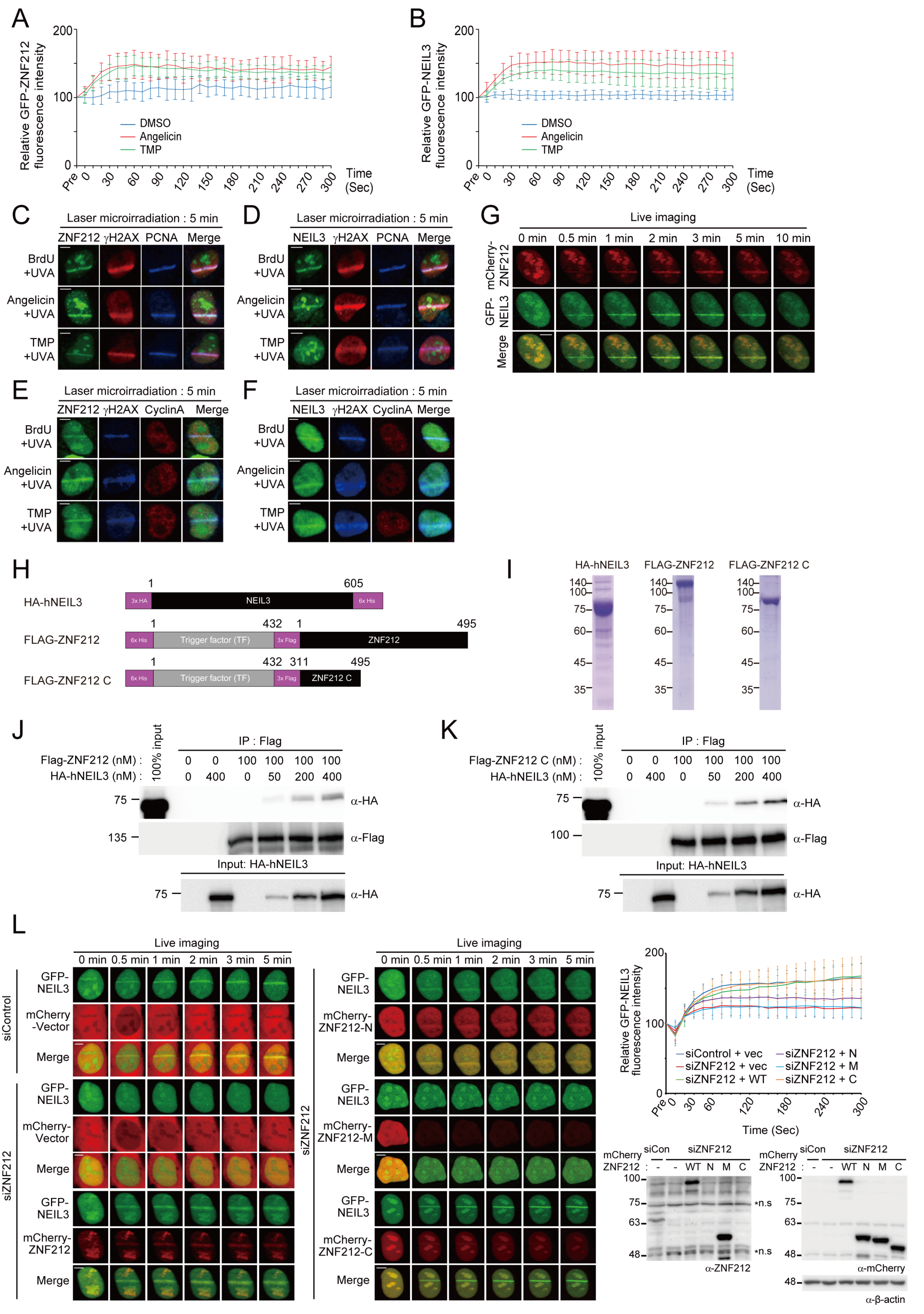

A
Relative GFP-ZNF212 fluorescence intensity
DMSO
Angelicin
TMP
Time (Sec)
B
Relative GFP-NEIL3 fluorescence intensity
DMSO
Angelicin
TMP
Time (Sec)
C
Laser microirradiation : 5 min
ZNF212 γH2AX PCNA Merge
BrdU +UVA
Angelicin +UVA
TMP +UVA
D
Laser microirradiation : 5 min
NEIL3 γH2AX PCNA Merge
BrdU +UVA
Angelicin +UVA
TMP +UVA
G
Live imaging
0 min 0.5 min 1 min 2 min 3 min 5 min 10 min
mCherry-ZNF212
GFP-NEIL3
Merge
E
Laser microirradiation : 5 min
ZNF212 γH2AX CyclinA Merge
BrdU +UVA
Angelicin +UVA
TMP +UVA
F
Laser microirradiation : 5 min
NEIL3 γH2AX CyclinA Merge
BrdU +UVA
Angelicin +UVA
TMP +UVA
H
HA-hNEIL3
3x HA NEIL3 6x His
1 605
FLAG-ZNF212
6x His Trigger factor (TF) 3x Flag ZNF212
1 432 1 495
FLAG-ZNF212 C
6x His Trigger factor (TF) 3x Flag ZNF212 C
1 432 311 495
I
HA-hNEIL3 FLAG-ZNF212 FLAG-ZNF212 C
J
100% input
IP : Flag
Flag-ZNF212 (nM) : 0 0 100 100 100 100
HA-hNEIL3 (nM) : 0 400 0 50 200 400
α-HA
α-Flag
Input: HA-hNEIL3
α-HA
K
100% input
IP : Flag
Flag-ZNF212 C (nM) : 0 0 100 100 100 100
HA-hNEIL3 (nM) : 0 400 0 50 200 400
α-HA
α-Flag
Input: HA-hNEIL3
α-HA
L
Live imaging
0 min 0.5 min 1 min 2 min 3 min 5 min
siControl
GFP-NEIL3
mCherry-Vector
Merge
siZNF212
GFP-NEIL3
mCherry-Vector
Merge
GFP-NEIL3
mCherry-ZNF212
Merge
Live imaging
0 min 0.5 min 1 min 2 min 3 min 5 min
siZNF212
GFP-NEIL3
mCherry-ZNF212-N
Merge
GFP-NEIL3
mCherry-ZNF212-M
Merge
GFP-NEIL3
mCherry-ZNF212-C
Merge
Relative GFP-NEIL3 fluorescence intensity
siControl + vec
siZNF212 + vec
siZNF212 + WT
siZNF212 + N
siZNF212 + M
siZNF212 + C
Time (Sec)
mCherry siCon siZNF212
ZNF212 : - - WT N M C
*n.s
*n.s
α-ZNF212
mCherry siCon siZNF212
ZNF212 : - - WT N M C
α-mCherry
α-β-actin

Supplementary Figure S12. ZNF212 promotes NEIL3 recruitment to DNA damage sites. (A - B) Relative intensity of GFP-ZNF212 (A) and GFP-NEIL3 (B) to laser stripes in presence of DMSO, Angelicin, or TMP. (C - F) U2OS cells were transfected with either GFP-ZNF212 (C and E) or GFP-NEIL3 (D and F). 24 hr post-transfection, transfected cells were treated with BrdU, angelicin or TMP, and then treated with laser microirradiation. 5 min after microirradiation, the cells were fixed and stained with indicated antibodies. (G) Upon laser microirradiation, the recruitment of GFP-NEIL3 and mCherry-ZNF212 translocation to DNA damage sites was monitored with live cell imaging at each indicated time. (H) Schematic illustration of bacterial expression vectors for hNEIL3, hZNF212-WT, and hZNF212-C. (I) The Coomassie blue stain shows the purified ZNF212 and NEIL3. (J and K) The direct association between NEIL3 and ZNF212 WT or C term mutant. (L) Co-transfection of GFP-NEIL3 with each of mCherry-vec, mCherry-ZNF212-WT, mCherry-ZNF212-N, mCherry-ZNF212-M, or mCherry-ZNF212-C mutant plasmid in siControl or siZNF212 transfected U2OS cells. After 24 hr, the transfected cells were microirradiated in the presence of TMP and recruitment of NEIL3 and ZNF212 to laser strips was examined by live cell imaging. The initial intensity of region of interest (ROI) before bleaching was calculated as 100% in each cell, and then the average intensity of the laser stripes was plotted (right upper panel). For each experiment, ten cells were analyzed. Data represent the mean ±SD from two independent experiments. Scale bar, 5 μm. Expression level for endogenous and exogenous ZNF212 were compared by Western blot analysis with indicated antibodies.

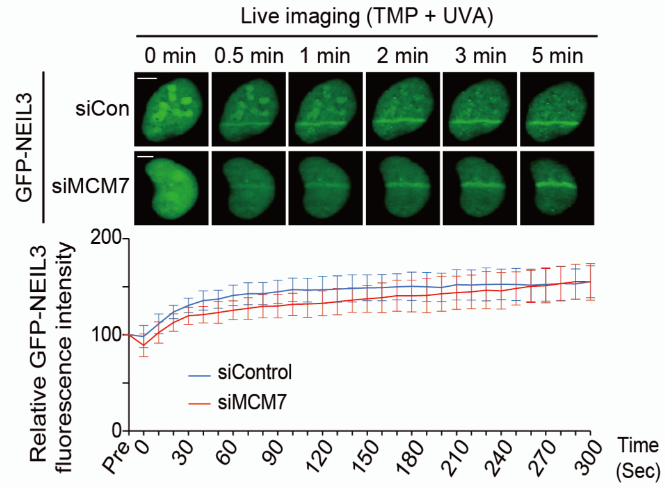

Supplementary Figure S13. Depletion of MCM7 does not significantly affect GFP-NEIL3 accumulation on the ICL damage sites. U2OS cells treated with either control or MCM7 siRNAs were transfected with GFP-NEIL3, and then treated with laser microirradiation 24 hr post-transfection in the presence of TMP. We examined the laser strip at each indicated time. The relative intensity of GFP-NEIL3 to the laser stripes from ten cells was quantified and summarized in graph. The initial intensity of region of interest (ROI) before bleaching was calculated as 100% in each cell, and then the average intensity of the laser stripes was plotted. Data represent the mean ± SD from two experiments. Scale bar, 5 μm.

Supplementary Table S1. The full list of oligonucleotides used in this study

Name	Sequences of oligonucleotide (5' to 3')
ZNF212_SFB_Myc_For	GCGCGCACCGGTGAATTCATGGCGGAGTCGGCGCCT
ZNF212_SFB_Myc_Rev	GCGCGCCTCGAGGTCGACTTAAAGCAGGCCATTGGG
ZNF212_eGFPC2_For	GCGCGCCTCGAGCATGGCGGAGTCGGCGCCTGCT
ZNF212_eGFPC2_Rev	GCGCGCGAATTCTTAAAGCAGGCCATTGGGCCTTCC
ZNF212_mCherryC1_For	GCGCGCTCGAGGCATGGCGGAGTCGGCGCCTGCT
ZNF212_mCherryC1_Rev	GCGCGCGAATTCTTAAAGCAGGCCATTGGGCCTTCC
ZNF212_N_For	GCGCGCCTCGAGCATGGCGGAGTCGGCGCCTGCT
ZNF212_N_Rev	GCGCGCGAATTCTTAACCTGCTGGGTGGGCACCACC
ZNF212_M_For (NLS)	GCGCGCGCCTCGAGCATGCCAAAAAAAAAAAGAAAAGTTGAAGGTCCTGGTG GTGCCCAC
ZNF212_M_Rev	GCGCGCGAATTCTTACTCAGAACATTCGTAGGGGCG
ZNF212_C_For	GCGCGCGCCTCGAGCATGGACACTTCCCGCCCCTACGAA
ZNF212_C_Rev	GCGCGCGAATTCTTAAAGCAGGCCATTGGGCCTTCC
ZNF212_D1_For	CAGACCACCGAGATTTCAGAGAACGTGGAGAACCTG
ZNF212_D1_Rev	CAGGTTCTCCACGTTCTCTGAAATCTCGGTGGTCTG
ZNF212_D2_For	GAGGCCCCCAAGGTGTCCGCCCACCCAGCAGGTGGG
ZNF212_D2_Rev	CCCACCTGCTGGGTGGGCGGACACCTTGGGGGCCTC
ZNF212_D3_For	GAGGAAGGTCCTGGTGGTTACGAATGTTCTGAGTGT
ZNF212_D3_Rev	ACACTCAGAACATTCGTAACCACCAGGACCTTCCTC
ZNF212_D4_For	AAGGACACTTCCCGCCCCTCTGGGTGGGGGTCTTGT
ZNF212_D4_Rev	ACAAGACCCCCACCCAGAGGGGCGGGAAGTGTCCTT
ZNF212_D5_For	ACCAAAAAGGCCAAGCTGCTGCAGGAGGGG CCCAGT
ZNF212_D5_Rev	ACTGGGCCCCTCCTGCAGCAGCTTGGCCTTTTTGGT
ZNF212_D6_For	AGGAAAAGCCGGAGTTCCACGGGTGAGCGGCCCTAC
ZNF212_D6_Rev	GTAGGGCCGCTCACCCGTGGAACTCCGGCTTTTCCT
ZNF212_D7_For	CACACGGGTGAGCGGCCCCAGCGGGAGCGGGGTGGG
ZNF212_D7_Rev	CCCACCCCGCTCCCGCTGGGGCCGCTCACCCGTGTG
ZNF212_siRNA_Resi_For	GCAGGTGGGGTCATGATTAAGCAAGAACTGCAATACACACAG GAAGGCCCT
ZNF212_siRNA_Resi_Rev	AGGGCCTTCCTGTGTGTATTGCAGTTCTTGCTTAATCATGAC CCCACCTGC
mTraip_Myc-His_For	ATATGGTACCATGCCTATCCGCGCTCTGTGCACTATC
mTraip_Myc-His_Rev	CACAGCGGCCGCCCCTGACATAAGAAGGTATCCAGCTTGGGCTG
mTraip_R18C_For	CACAGGTACCATGCCTATCCGCGCTCTGTGCACTATCTGCTCCGACTTCTTCG ATCACTCCTGTGACGTGGC
mTraip_R18C_Rev	CACAGCGGCCGCCCCTGACATAAGAAGGTATCCAGCTTGGGCTG
mTraip_D1_For	CCTATCCGCGCTCTGTGCGTTGGCAAAAAGACTATT
mTraip_D1_Rev	AATAGTCTTTTTGCCAACGCACAGAGCGCGGATAGG
mTraip_D5_For	ATATGGTACCATGCCTATCCGCGCTCTGTGCACTATC
mTraip_D5_Rev	ACACACGCGGCCGCCCGGAGGCAGAACTCACAGT
mTraip_dPIP_For	ATATGGTACCATGCCTATCCGCGCTCTGTGCACTATC
mTraip_dPIP_Rev	ACACACGCGGCCGCCCGGAGGCAGAACTCACAGT
mTraip_D5+PIP_For	ACAGATGTGGTAAGAATACAGCCCAAGCTGGATACC
mTraip_D5+PIP_Rev	GGTATCCAGCTTGGGCTGTATTCTTACCACATCTGT

NEIL3_eGFPN1_For	ATATGAATTCACCGGTATGGTGGAAGGACCAGGCTGTACTC
NEIL3_eGFPN1_Rev	GCGCGCGGATCCGGGCATCCAGGAATAATTTTTAT
NEIL3_Myc_For	ATATGAATTCACCGGTATGGTGGAAGGACCAGGCTGTACTC
NEIL3_Myc_Rev	AGAGCTCGAGTTAGCATCCAGGAATAATTTTTATTCC
Mouse Zfp212 guide RNA #1 For	CACC**G**TGTGGCTGGAGCTAATGGCG
Mouse Zfp212 guide RNA #1 Rev	AAACCGCCATTAGCTCCAGCCACA**C**
Mouse Zfp212 guide RNA #2 For	CACC**G**GTGGCTGCTATTCAAGCCG
Mouse Zfp212 guide RNA #2 Rev	AAACCGGCTTGAATAGCAGCCAC**C**
Mouse Traip guide RNA #1 For	CACC**G**CTTCGATCACTCCCGTGACG
Mouse Traip guide RNA #1 Rev	AAACCGTCACGGGAGTGATCGAAG**C**
Mouse Traip guide RNA #2 For	CACC**G**TGGCTCGACTCAAGCAGCCC
Mouse Traip guide RNA #2 Rev	AAACGGGCTGCTTGAGTCGAGCCA**C**
Mouse Neil3 guide RNA #1 For	CACC**G**ATCTTGATTAACCCACGGGA
Mouse Neil3 guide RNA #1 Rev	AAACTCCCGTGGGTTAATCAAGAT**C**
Mouse Neil3 guide RNA #2 For	CACC**G**TCTGGTGAGCTGCACCGCCA
Mouse Neil3 guide RNA #2 Rev	AAACTGGCGGTGCAGCTCACCAGA**C**
Mouse Fancd2 guide RNA #1 For	CACC**G**TTCCGCCATGATGCCGGCCA
Mouse Fancd2 guide RNA #1 Rev	AAACTGGCCGGCATCATGGCGGAA**C**
Mouse Fancd2 guide RNA #2 For	CACC**G**ACGACATGCACCTGGTGATC
Mouse Fancd2 guide RNA #2 Rev	AAACGATCACCAGGTGCATGTCGT**C**
Mouse Zfp212 sequencing DF	ACACGATATCTAACCAGTACACTGTCTGAATG
Mouse Zfp212 sequencing DR	ACACGCGGCCGCTTCTTGTTTCGAAGCAGGTTCTC
Mouse Traip sequencing DF	ACACGATATCAAGGAGCTAAATCCCAGGATTC
Mouse Traip sequencing DR	ACACGCGGCCGCAAAGAAGCACTCTTTCACCGTGG
Mouse Neil3 sequencing DF	ACACGATATCTTCAGACAAAACAGGTAGG
Mouse Neil3 sequencing DR	ACACGCGGCCGCAAGCAACATGTGTGATGTTG
Mouse Fancd2 sequencing DF	ACACGATATCTTCTCAAGTCCTAGAGCAGTTG
Mouse Fancd2 sequencing DR	ACACGCGGCCGCAAAGTGTGTAAGGTGAATGGG
ZNF212 siRNA #1	CCUUCACAAGCAACAGAGAUU
ZNF212 siRNA #2	CAAACAGGAGCUACAGUAUUU
ZNF212 siRNA #3	CCUCAUCUGUGGUUACUGUUU
ZNF212 siRNA #4	CAUUCAUGCCAAUGCUUGUUU
ZNF212 siRNA #5	CUUUGUUUAAUAAUAAGUAUU
TRAIP siRNA	GCAAGUUGCAGACAGUCUAUU
NEIL3 siRNA #1	GGGUGGAUCAUGUUAUGGAUU
NEIL3 siRNA #2	GCUAAUGGAUCAGAACGUAUU
RAD51 siRNA	On-TARGETplus SMARTpool-Human (Dharmacon, RAD51Cat#L-003530-00)

Supplementary Table S2. The list of hits identified from TRAIP Y2H screening

Prey Description			Reporter expression			Number of clones
Protein	Description	Accession number	lacZ	URA3	ADE2	
KRT18	keratin 18, transcript variant 2	NM_199187	-	+	+	6
	keratin 18, transcript variant 1	NM_000224	-	+	+	5
SPAG5	sperm associated antigen 5	NM_006461	+	+	+	10
VIM	vimentin	NM_003380	-	+	+	7
			+	+	+	1
ZNF212	zinc finger protein 212	NM_012256	+	+	+	6
TPM3	tropomyosin 3, transcript variant Tpm3.1	NM_153649	+	+	+	5
PSME3	proteasome activator subunit 3, transcript variant 1	NM_005789	+	+	+	5
UIMC1	ubiquitin interaction motif containing 1, transcript variant 2	NM_016290	-	+	+	4
HNRNPK	heterogeneous nuclear ribonucleoprotein K, transcript variant 1	NM_002140	-	+	+	3
TRIM23	tripartite motif containing 23, transcript variant gamma	NM_033228	+	+	+	3
TRIM27	tripartite motif containing 27	NM_006510	+	+	+	2
TPR	translocated promoter region, nuclear basket protein	NM_003292	+	+	+	2
RNF20	ring finger protein 20, E3 ubiquitin protein ligase	NM_019592	-	+	+	2
EFEMP1	EGF containing fibulin-like extracellular matrix protein 1, transcript variant 3	NM_001039349	-	+	+	2
CEP72	centrosomal protein 72, transcript variant 1	NM_018140	+	+	+	2
CCHCR1	coiled-coil alpha-helical rod protein 1, transcript variant 1	NM_001105564	+	+	+	2
KRT10	keratin 10, transcript variant 1	NM_000421	+	+	+	1
			-	+	+	1
TNIP2	TNFAIP3 interacting protein 2, transcript variant 1	NM_024309	+	+	+	1
	TNFAIP3 interacting protein 2, transcript variant 2	NM_001161527	+	+	+	1
TNIP1	TNFAIP3 interacting protein 1, transcript variant 10	NM_001258456	-	+	+	1
TSG101	tumor susceptibility gene 101	NM_006292	+	+	+	1
TMX2	thioredoxin-related transmembrane protein 2, transcript variant 2	NM_001144012	-	+	+	1
TAB3	TGF-beta activated kinase 1/MAP3K7 binding protein 3	NM_001105564	+	+	+	1
PPP1R12C	protein phosphatase 1, regulatory subunit 12C, transcript variant 1	NM_017607	-	+	+	1
PPHLN1	periphilin 1, transcript variant 8	NM_001143789	+	+	+	1
PIAS1	protein inhibitor of activated STAT 1, transcript variant 2	NM_016166	+	+	+	1
PGK1	phosphoglycerate kinase 1	NM_000291	-	+	+	1
MIF4GD	MIF4G domain containing, transcript variant 3	NM_001242500	+	+	+	1
LZTS2	leucine zipper, putative tumor suppressor 2	NM_032429	+	+	+	1
HNRNPC	heterogeneous nuclear ribonucleoprotein C (C1/C2), transcript variant 4	NM_001077443	-	+	+	1
GOLGB1	golgin B1, transcript variant 4	NM_001256488	+	+	+	1
COG6	component of oligomeric golgi complex 6, transcript variant 2	NM_001145079	-	+	+	1
CNTROB	centrobin, centrosomal BRCA2 interacting protein, transcript variant 1	NM_053051	+	+	+	1
CDC37	cell division cycle 37	NM_007065	-	+	+	1
CCDC14	coiled-coil domain containing 14, transcript variant 1	NM_022757	-	+	+	1
CASP8AP2	caspase 8 associated protein 2, transcript variant 2	NM_001137667	+	+	+	1

Supplementary Table S3. The sequence information for mutant alleles in knockout mESCs

Gene	Allele	Deleted DNA sequences in mouse exon (in red) and intron (in blue)	Size of deletion	comment
Traip	WT allele (exon 1)	5'-<br>ACTACCGGAAGTCGTGCGGTGGAGCGAAATTTGAAGGAACCGGAGCGGT<br>GGCCGGTTCGGCCACCAAACTGTGTCTGTCGCTGGCAGCTGGTTCCCTG<br>GGCTGCTTGAGTCGAGCCATCATGCCTATCCGCGCTCTGTGCACTATCTG<br>CTCCGACTTCTTCGATCACTCCCGTGACGTGGCTGCCATCCACTGTGGCC<br>ACACTTTTCATCTGCAATG-3'	Wild type	
Traip KO #2	Mutant allele 1 & 2	5'-<br>ACTACCGGAAGTCGTGCGGTGGAGCGAAATTTGAAGGAACCGGAGCGGT<br>GGCCGGTTCGGCCACCAAACTGTGTCTGTCGCTGGCAGCTGGTTCCCTG<br>GGCTGCTTGAGTCGAGCCATCATGCCTATCCGCGCTCTGTGCACTATCTG<br>CTCCGACTTCTTCGATCACTCCCGTGACGTGGCTGCCATCCACTGTGGCC<br>ACACTTTTCATCTGCAATG-3'	74bp del	
Traip KO #11	Mutant allele 1	5'-<br>ACTACCGGAAGTCGTGCGGTGGAGCGAAATTTGAAGGAACCGGAGCGGT<br>GGCCGGTTCGGCCACCAAACTGTGTCTGTCGCTGGCAGCTGGTTCCCTG<br>GGCTGCTTGAGTCGAGCCATCATGCCTATCCGCGCTCTGTGCACTATCTG<br>CTCCGACTTCTTCGATCACTCCCGTGACGTGGCTGCCATCCACTGTGGCC<br>ACACTTTTCATCTGCAATG-3'	5bp del	
	Mutant allele 2	5'-<br>ACTACCGGAAGTCGTGCGGTGGAGCGAAATTTGAAGGAACCGGAGCGGT<br>GGCCGGTTCGGCCACCAAACTGTGTCTGTCGCTGGCAGCTGGTTCCCTG<br>GGCTGCTTGAGTCGAGCCATCATGCCTATCCGCGCTCTGTGCACTATCTG<br>CTCCGACTTCTTCGATCACTCCCGTGACGTGGATGGCAGCCACTGTGGC<br>CACACTTTTCATCTGCAATG-3'	5bp del	C->A, C->G, C-G substitution
Zfp212-Traip DKO #8	Mutant allele 1 &2	5'-<br>ACTACCGGAAGTCGTGCGGTGGAGCGAAATTTGAAGGAACCGGAGCGGT<br>GGCCGGTTCGGCCACCAAACTGTGTCTGTCGCTGGCAGCTGGTTCCCTG<br>GGCTGCTTGAGTCGAGCCATCATGCCTATCCGCGCTCTGTGCACTATCTG<br>CTCCGACTTCTTCGATCACTCCCGTGACGTGGCTGCCATCCACTGTGGCC<br>ACACTTTTCATCTGCAATG-3'	2bp del	104bp Del (5' UTR)
Fancb-Traip DKO #1	Mutant allele 1	5'-<br>ACTACCGGAAGTCGTGCGGTGGAGCGAAATTTGAAGGAACCGGAGCGGT<br>GGCCGGTTCGGCCACCAAACTGTGTCTGTCGCTGGCAGCTGGTTCCCTG<br>GGCTGCTTGAGTCGAGCCATCATGCCTATCCGCGCTCTGTGCACTATCTG<br>CTCCGACTTCTTCGATCACTCCCGTGACGTGGCTGCCATCCACTGTGGCC<br>ACACTTTTCATCTGCAATG-3'	10bp del	
	Mutant allele 2	5'-<br>ACTACCGGAAGTCGTGCGGTGGAGCGAAATTTGAAGGAACCGGAGCGGT<br>GGCCGGTTCGGCCACCAAACTGTGTCTGTCGCTGGCAGCTGGTTCCCTG<br>GGCTGCTTGAGTCGAGCCATCATGCCTATCCGCGCTCTGTGCACTATCTG<br>CTCCGACTTCTTCGATCACTCCCGTGACGTGGCTGCCATCCACTGTGGCC<br>ACACTTTTCATCTGCAATG-3'	50bp del	
Zfp212	WT allele (exon 2)	5'-<br>GCACAGGAGGAAACGGCGCGCCACGCCATTAGCTCCAGCCACACCACCT<br>TCACAGACAACAGAGAAAAGCCCGTACTTTCAGACCACAGAAATCTCACT<br>TTGGACAGTGGTGGCTGCTATTCAAGCCGTGGAGAA-3'	Wild type	
Zfp212 KO #7	Mutant allele 1	5'-<br>GCACAGGAGGAAACGGCGCGCCACGCCATTAGCTCCAGCCACACCACCT<br>TCACAGACAACAGAGAAAAGCCCGTACTTTCAGACCACAGAAATCTCACT<br>TTGGACAGTGGTGGCTGCTATTCAAGCCGTGGAGAA-3'	14 (11 + 3)bp del	
	Mutant allele 2	5'-<br>GCACAGGAGGAAACGGCGCGCCACGCCATTAGCTCCAGCCACACCACCT<br>TCACAGACAACAGAGAAAAGCCCGTACTTTCAGACCACAGAAATCTCACT<br>TTGGACAGTGGTGGCTGCTATTCAAGCCGTGGAGAA-3'	43bp del	
Zfp212 KO #20	Mutant allele 1 & 2	5'-<br>GCACAGGAGGAAACGGCGCGCCACTCCATTAGCTCCAGCCACACCACCT<br>TCACAGACAACAGAGAAAAGCCCGTACTTTCAGACCACAGAAATCTCACT<br>TTGGACAGTGGTGGCTGCTATTCAAGCCGTGGAGAA-3'	14 (5+3+1+ 1+1+1+2)bp del	
Fancb-Zfp212 DKO #13	Mutant allele 1 & 2	5'-<br>GCACAGGAGGAAACGGCGCGCCACGCCATTAGCTCCAGCCACACCACCT<br>TCACAGACAACAGAGAAAAGCCCGTACTTTCAGACCACAGAAATCTCACT<br>TTGGACAGTGGTGGCTGCTATTCAAGCCGTGGAGAAG-3'	4 (1+ 3)bp del	
Fancb-Zfp212 DKO #14	Mutant allele 1	5'-<br>GCACAGGAGGAAACGGCGCGCCACGCCATTAGCTCCAGCCACACCACCT<br>TCACAGACAACAGAGAAAAGCCCGTACTTTCAGACCACAGAAATCTCACT<br>TTGGACAGTGGTGGCTGCTATTCAAGCCGTGGAGAAG-3'	4 (1+ 3)bp del	
	Mutant allele 2	5'-<br>GCACAGGAGGAAACGGCGCGCCACGCCATTAGCTCCAGCCACACCACCT<br>TCACAGACAACAGAGAAAAGCCCGTACTTTCAGACCACAGAAATCTCACT<br>TTGGACAGTGGTGGCTGCTATTCAAGCCGTGGAGAAGA-3'	5 (3 + 2)bp del	
Neil3	WT allele (exon 3)	5'-<br>GATTCATTTCGGAATGAAAGGCTCCATCTTGATTAACCCACGGGAGGGTG<br>AGAACAGAGCTGGGGCTTCTCCAGCCTTGGCGGTGCAGCTCACCAGAGA<br>CTTGATCTGCTTCTATGACTCTTCAGTAGAACTCAG-3'	Wild type	
Neil3 KO #10	Mutant allele 1 & 2	5'-<br>GATTCATTTCGGAATGAAAGGCTCCATCTTGATTAACCCACGGGAGGGTG<br>AGAACAGAGCTGGGGCTTCTCCAGCCTTGGCGGTGCAGCTCACCAGAGA<br>CTTGATCTGCTTCTATGACTCTTCAGTAGAACTCAG -3'	40bp del	
Neil3 KO #19	Mutant allele 1 & 2	5'-<br>GATTCATTTCGGAATGAAAGGCTCCATCTTGATTAACCCACGGGAGGGTG<br>AGAACAGAGCTGGGGCTTCTCCAGCCTTGGCGGTGCAGCTCACCAGAGA<br>CTTGATCTGCTTCTATGACTCTTCAGTAGAACTCAG -3'	38bp del	
Zfp212-Neil3 DKO #1	Mutant allele 1	5'-<br>TACCATTTTTAAAAAGAAAATAAAATGATGACACATGATCTGCTTCTGTTAC<br>TCAAAAATGGGCTGGCTCTGTTTCAGGATTCATTTCGGAATGAAAGGCTC<br>CATCTTGATTAACCCACGGGAGGGTGAGAACAGAGCTGGGGCTTCTCCA<br>GCCTTGGCGGTGCAGCTCACCAGAGACTTGATCTGCTTCTATGACTCTTC<br>AGTAGAACTCAG-3'	135bp del	78bp del (intron 2)

	Mutant allele 2	5'-CAAAAATGGGCTGGCTCTGTTTCAGGATTCATTTCGGAATGAAAGGCTCCATCTTGATTAACCCACGGGAGGGTGAGAACAGAGCTGGGGCTTCTCCAGCCTTGGCGGTGCAGCTCACGGTGCAGCTCACCAGAGACTTGATCTGCTTCTATGACTCTTCAGTAGAACTCAG-3'	92bp del	25bp del (intron 2)
Zfp212-Neil3 DKO #37	Mutant allele 1	5'-TACCATTTTTAAAAAGAAAATAAAATGATGACACATGATCTGCTTCTGTTACTCAAAAATGGGCTGGCTCTGTTTCAGGATTCATTTCGGAATGAAAGGCTCCATCTTGATTAACCCACGGGAGGGTGAGAACAGAGCTGGGGCTTCTCCAGCCTTGGCGGTGCAGCTCACCAGAGACTTGATCTGCTTCTATGACTCTTCAGTAGAACTCAG-3'	135bp del	78bp del (intron 2)
	Mutant allele 2	5'-GATTCATTTCGGAATGAAAGGCTCCATCTTGATTAACCCACGGGAGGGTGAGAACAGAGCTGGGGCTTCTCCAGCCTTGGCGGTGCAGCTCACCAGAGACTTGATCTGCTTCTATGACTCTTCAGTAGAACTCAG-3'	10bp del	
Traip-Neil3 DKO #18	Mutant allele 1	5'-GATTCATTTCGGAATGAAAGGCTCCATCTTGATTAACCCACGGGAGGGTGAGAACAGAGCTGGGGCTTCTCCAGCCTTGGCGGTGCAGCTCACCAGAGACTTGATCTGCTTCTATGACTCTTCAGTAGAACTCAG-3'	14 (13 + 1)bp del	
	Mutant allele 2	5'-GATTCATTTCGGAATGAAAGGCTCCATCTTGATTAACCCACGGGAGGGTGAGAACAGAGCTGGGGCTTCTCCAGCCTTGGCGGTGCAGCTCACCAGAGACTTGATCTGCTTCTATGACTCTTCAGTAGAACTCAG-3'	52bp del	
Traip-Neil3 DKO #19	Mutant allele 1 & 2	5'-GATTCATTTCGGAATGAAAGGCTCCATCTTGATTAACCCACGGGAGGGTGAGAACAGAGCTGGGGCTTCTCCAGCCTTGGCGGTGCAGCTCACCAGAGACTTGATCTGCTTCTATGACTCTTCAGTAGAACTCAG-3'	38bp del	
Fancb-Neil3 DKO #1	Mutant allele 1	5'-GATTCATTTCGGAATGAAAGGCTCCATCTTGATTAACCCACGGGAGGGTGAGAACAGAGCTGGGGCTTCTCCAGCCTTGGCGGTGCAGCTCACCAGAGACTTGATCTGCTTCTATGACTCTTCAGTAGAACTCAG -3'	40bp del	
	Mutant allele 2	5'-GATTCATTTCGGAATGAAAGGCTCCATCTTGATTAACCCACGGGAGGGTGAGAACAGAGCTGGGGCTTCTCCAGCCTTGGCGGTGCAGCTCACCAGAGACTTGATCTGCTTCTATGACTCTTCAGTAGAACTCAG-3'	38bp del	
Fancb-Neil3 DKO #13	Mutant allele 1 & 2	5'-GATTCATTTCGGAATGAAAGGCTCCATCTTGATTAACCCACGGGAGGGTGAGAACAGAGCTGGGGCTTCTCCAGCCTTGGCGGTGCAGCTCACCAGAGACTTGATCTGCTTCTATGACTCTTCAGTAGAACTCAG-3'	38 (24 + 14) bp del	
Fancb-Neil3 DKO #27	Mutant allele 1 & 2	5'-GATTCATTTCGGAATGAAAGGCTCCATCTTGATTAACCCACGGGAGGGTGAGAACAGAGCTGGGGCTTCTCCAGCCTTGGCGGTGCAGCTCACCAGAGACTTGATCTGCTTCTATGACTCTTCAGTAGAACTCAG-3'	38bp del	
Fancd2	WT allele (exon 18)	5'-GACGACATGCACCTGGTGATCCGGAAGCAGCTCTCTAGCACTGTGTTCAAGTACAAGCTCATTGGGATCATTGGTGCAGTCACCATGGCCGGCATCATGGCGGAAGACAG-3'	Wild type	
Fancd2 KO #18	Mutant allele 1 & 2	5'-GACGACATGCACCTGGTGATCCGGAAGCAGCTCTCTAGCACTGTGTTCAAGTACAAGCTCATTGGGATCATTGGTGCAGTCACCATGGCCGGCATCATGGCGGAAGACAG-3'	70 (2 +68)bp del	
Fancd2-Zfp212 DKO #13	Mutant allele 1	5'-GACGACATGCACCTGGTGATCCGGAAGCAGCTCTCTAGCACTGTGTTCAAGTACAAGCTCATTGGGATCATTGGTGCAGTCACCATGGCCGGCATCATGGCGGAAGACAG-3'	100bp del	
	Mutant allele 2	5'-GACGACATGCACCTGGTGATCCGGAAGCAGCTCTCTAGCACTGTGTTCAAGTACAAGCTCATTGGGATCATTGGTGCAGTCACCATGGCCGGCATCATGGCGGAAGACAG-3'	70 (2 + 68)bp del	

Supplementary Table S4. Mean value (upper) and statistics (p value, bottom) for MMC-induced and spontaneous chromosomal abnormalities.

Mean value	MMC (30 nM)			DMSO (Spontaneous)		
	DNA breaks	Radials	Abnormal Chrom.	DNA breaks	Radials	Abnormal Chrom.
**AB2.2**	0.4571	0.0000	0.4571	0.1143	0.0000	0.1143
**Traip KO**	1.7143	0.0286	1.7429	0.2286	0.0000	0.2286
**Zfp212 KO**	0.8571	0.0286	0.8857	0.1714	0.0000	0.1714
**ZT-DKO**	2.8571	0.3143	3.1714	0.4000	0.0000	0.4000

p value (AB vs)		Zfp212 KO	Traip KO	ZT-DKO
**MMC**	**DNA breaks**	0.1061	<0.0001	0.0018
	**Radials**	0.3209	0.3209	0.0008
	**Abnormal chrom.**	0.1076	<0.0001	0.0006
**DMSO**	**DNA breaks**	0.5453	0.2102	0.0103
	**Radials**	NA	NA	NA
	**Abnormal chrom.**	0.5453	0.2102	0.0103

Supplementary Table S5. Mean value (upper) and statistics (p value, bottom) for TMP-induced, MMC-induced and spontaneous chromosomal abnormalities.

Mean value	TMP			MMC			DMSO		
	DNA breaks	Radials	Abnormal Chrom.	DNA breaks	Radials	Abnormal Chrom.	DNA breaks	Radials	Abnormal Chrom.
AB2.2	0.1143	0.0000	0.1143	0.1143	0.0000	0.1143	0.0571	0.0000	0.0571
Traip KO	0.2857	0.0571	0.3429	0.2000	0.0000	0.2000	0.2000	0.0000	0.2000
Zfp212 KO	0.3143	0.0286	0.3429	0.0571	0.0000	0.0571	0.0857	0.0000	0.0857
Fancb KO	1.7143	0.2286	2.0857	1.8286	0.3143	2.1429	0.3143	0.0000	0.3143
Neil3 KO	0.5714	0.2286	0.8000	0.2857	0.0000	0.2857	0.0000	0.0000	0.0000
TN-DKO	0.7429	0.1714	0.9143	0.2571	0.0000	0.2571	0.1429	0.0000	0.1429
ZN-DKO	0.5429	0.1429	0.6857	0.3143	0.0000	0.3143	0.0571	0.0000	0.0571
FT-DKO	5.8286	0.3714	6.2000	4.4000	0.3429	4.7429	1.2000	0.0286	1.2286
FZ-DKO	4.6286	0.4000	5.0286	1.8857	0.1429	2.0286	0.7714	0.0286	0.8000
FN-DKO	6.4857	0.6286	7.1143	5.4857	0.2857	5.7714	1.0000	0.0286	1.0286

p value (AB vs)		Traip KO	Zfp212 KO	Fancb KO	Neil3 KO	TN-DKO	ZN-DKO	FT-DKO	FZ-DKO	FN-DKO
TMP	DNA breaks	0.1523	0.0802	<0.0001	0.002	0.0001	0.0527	<0.0001	<0.0001	<0.0001
	Radials	0.1557	0.3209	0.0005	0.0159	0.0284	0.0201	<0.0001	<0.0001	<0.0001
	Abnormal chrom.	0.0632	0.0632	<0.0001	0.0002	<0.0001	0.0125	<0.0001	<0.0001	<0.0001
MMC	DNA breaks	0.3316	0.4005	<0.0001	0.0749	0.1633	0.0607	<0.0001	<0.0001	<0.0001
	Radials	NA	NA	0.0002	NA	NA	NA	<0.0001	<0.0001	<0.0001
	Abnormal chrom.	0.3316	0.4005	<0.0001	0.0749	0.1633	0.0607	<0.0001	<0.0001	<0.0001
DMSO	DNA breaks	0.0761	0.6483	0.0372	0.1557	0.2381	>0.9999	<0.0001	0.0002	<0.0001
	Radials	NA	NA	NA	NA	NA	NA	0.0037	0.0147	0.1557
	Abnormal chrom.	0.0761	0.6483	0.0372	0.1557	0.2381	>0.9999	<0.0001	<0.0001	<0.0001
